# Matheuristics for Multi-UAV Routing and Recharge Station Location for Complete Area Coverage

**DOI:** 10.3390/s21051705

**Published:** 2021-03-02

**Authors:** Rafael Santin, Luciana Assis, Alessandro Vivas, Luciano C. A. Pimenta

**Affiliations:** 1 Department of Computing, Universidade Federal dos Vales do Jequitinhonha e Mucuri, Rod. MGT 367, Km 583, 5000-Alto da Jacuba, Diamantina 39100-000, Brazil; lpassis@ufvjm.edu.br (L.A.); alessandrovivas@ufvjm.edu.br (A.V.); 2 Graduate Program in Electrical Engineering, Universidade Federal de Minas Gerais, Av. Antônio Carlos 6627, Belo Horizonte 31270-901, Brazil; 3 Department of Electronic Engineering, Universidade Federal de Minas Gerais, Belo Horizonte 31270-901, Brazil; lucpim@cpdee.ufmg.br

**Keywords:** area coverage, multi-UAVs routing problem, multiobjective optimization, matheuristics, charging location

## Abstract

This paper presents matheuristics for routing a heterogeneous group of capacitated unmanned air vehicles (UAVs) for complete coverage of ground areas, considering simultaneous minimization of the coverage time and locating the minimal number of refueling stations. Whereas coverage path planning (CPP) is widely studied in the literature, previous works did not combine heterogeneous vehicle performance and complete area coverage constraints to optimize UAV tours by considering both objectives. As this problem cannot be easily solved, we designed high-level path planning that combines the multiobjective variable neighborhood search (MOVNS) metaheuristic and the exact mathematical formulation to explore the set of nondominated solutions. Since the exact method can interact in different ways with MOVNS, we evaluated four different strategies using four metrics: execution time, coverage, cardinality, and hypervolume. The experimental results show that applying the exact method as an intraroute operator into the variable neighborhood descent (VND) can return solutions as good as those obtained by the closest to optimal strategy but with higher efficiency.

## 1. Introduction

As mobile sensing systems, unmanned aerial vehicles (UAVs) are a fast and efficient option to perceive the environment due to their quick response capabilities. The UAVs can assume autonomous behaviors performing complex tasks at a low operational cost, providing strong motivations to adopt these vehicles for various activities such as environmental monitoring, search and rescue activities, and precision agriculture.

Despite the remarkable benefits of using UAVs in such applications, it is important to highlight inherent limitations of the different UAV platforms, which must be considered when planning paths to cover a target area. Nowadays, the UAVs are classified as fixed-wing, rotary-wing (single and multirotor), and hybrids. Fixed-wing UAVs are characterized by their long endurance and fast flight speed, suitable for extensive area coverage applications such as monitoring power lines, roads, and pipelines. This UAV type requires some setup time to be launched, as this process usually has to be performed manually [1]. Some models are electric with four hours of endurance [2], and others use a fuel-injected engine that extends the flight time to up to 24 h [3]. However, they cannot perform vertical take-off and landing (VTOL). The multirotors are popular due to their maneuverability, hovering, and VTOL capability, but their low endurance usually restricts their flight range. The most common multirotor UAVs use battery technology that allows flights of 20 to 30 min, reaching 75 min on more advanced models [4]. The single-rotors have VTOL capability as do multirotors, but they are more efficient with longer endurance and greater payload capacity. Their power source can be combustion engines, allowing some models to fly for up to 4.8 h [5]. The hybrid UAVs combine the characteristics of fixed- and rotary-wing UAVs, such as the tilt-rotor-like aircraft. This configuration allows tilting the rotors to achieve the same VTOL capability as rotary-wing UAVs or flight dynamics as the fixed-wing, significantly improving its endurance [6]. Another hybrid configuration is a fixed-wing UAV with dedicated rotors to perform VTOL. An electric example of fixed-wing hybrid VTOL can achieve 2.5 h of flight time [7]. Notwithstanding the various types of UAVs and their performance differences, this paper proposes a high-level method that is able to plan coverage routes for all such types in a heterogeneous fleet. By properly setting up the input parameters of our method, the constraints inherent to each type of UAV can be incorporated.

In a different direction, some current solutions for route planning in coverage tasks tend to mitigate the limitations of using a particular UAV. An area decomposition strategy to generate paths with the minimum number of turns for fixed-wing UAVs, aiming to maximize coverage capability, was explored by Avellar et al. [1]. Since this type of UAV is manually prepared and launched, the authors proposed a mission planning methodology that considers human operator setup time capacity to adjust the number of vehicles to be deployed to minimize the total mission time.

A solution for planning routes to complete coverage using multirotors is presented in [8]. Unlike the decomposition strategy adopted in [1], the grid pattern map decomposition used by the authors cannot ensure a minimal number of turns. Each vehicle is assigned to a base where its mission starts and ends and its battery is recharged. As the bases can be installed in a set of cells predefined as potential bases, this strategy defines the path and the cell locations for opening the bases that minimize the maximum travel time for all UAVs.

The possibility of recharging the UAVs in different cells of the grid is presented in [9]. However, different from [8], the routing problem was not explored. In this case, the proposed method searches for the open paths that connect all grid cells, locating the recharging stations to minimize the vehicle’s energy consumption and the time to cover some high priority cells. Although this previous method was designed for long-term complete area coverage, it cannot be applied to planning missions that require the return of all vehicles to the base in a set minimum time, which is common in monitoring missions in areas without network infrastructure. In this case, the mission finishes when the sensing data are gathered after all vehicles return to the base station. An example is the protected areas in the Brazilian Amazon forest, which are remote regions without network coverage and with limited access to electricity.

Assunção et al. [10] indicated that remote-sensing-based monitoring and law enforcement significantly impact reducing the Amazon’s deforestation. Although using satellite imagery to identify forest cover changes provided significant advances in monitoring, this system has technical limitations, such as the lack of image resolution to identify forest losses in contiguous areas of less than 25 hectares. The study indicated a possible change in strategy to escape detection by the increase in the deforestation of small areas [11,12,13].

Thus, to improve the Brazilian environmental command and control strategy, the development of strategies, which can be used to plan fast and effective monitoring operations supplying the governmental authorities with precise information to identify and control small-scale deforestation and forest degradation, must be promoted [14]. Planning strategies for this kind of operation must ensure that the entire area is completely covered as quickly as possible, as detecting such damage earlier is critical for combat teams to act more effectively on site. In this case, the cellular decomposition method used in [1] is more appropriate since it ensures complete area coverage with an exact representation, reduces the UAV’s energy expenditure in the turns, and requires fewer vertices to represent the area than grid-based methods.

In this paper, we adopt the same area decomposition strategy as explored earlier by our team [1] and propose extending the previous path planning by presenting a matheuristic to deal with long-term coverage applications and heterogeneous UAVs. We design a high-level path planning method that defines the UAV routes for the coverage task and the recharging stations to be opened. As a high-level planner, the UAV particularities of fixed- and rotary-wing vehicles such as refueling time, curvature restriction, coverage altitude, the impact of take-off and landing method (autonomously or manually), and camera parameters are abstracted from the path planning method. In this case, all low-level factors must be mapped to the application by setting the proposed method’s input parameters adequately for each vehicle, such as flight time, speed, and the recharging ratio.

Due to the high costs of installing the recharging stations in the preservation areas, this high-level planner aims to minimize both the longest route among all the routes planned for the team and the number of necessary recharging stations, locating the places to open such facilities. Note that both minimization objectives (the longest flight time and the number of recharge stations) can be conflicting. Decreasing the number of recharging stations can force a UAV to make a detour to recharge, thereby increasing its coverage time.

The applied decomposition method represents each coverage row by a pair of vertices at its extremities. As the vehicle must visit these vertices, we use their positions as references to the recharging station locations. A conventional methodology to find the optimal solution for this multiobjective problem is to reduce it to a sequence of mono-optimization problems that can be modeled and theoretically solved by a commercial solver. However, the costs to compute a proven optimal solution is prohibitive in practice since this routing problem is known to be nondeterministic polynomial-time (NP)-hard.

We propose and evaluate four matheuristics distinguished by different forms of interactions between the multiobjective variable neighborhood search (MOVNS) metaheuristic and the exact method to solve this problem. The first matheuristic implemented uses the MOVNS to divide the coverage area among the UAVs and apply the exact method to build their routes. Every modification performed by the MOVNS is rerouted and evaluated to check if an improvement is obtained, which results in a high call rate of the exact method. This issue was mitigated by adopting heuristics to rearrange the route on the MOVNS and perform the route evaluation regardless of the exact method. One of these uses the exact method to build only the initial solution, and the other two strategies apply it to improve the solution at specific steps in the method. To evaluate the effect of these modifications, we analyzed the algorithm’s performance considering the computation time to return its solutions and their quality regarding the coverage, cardinality, and hypervolume metrics.

The main contributions of this paper are: (1) presenting a multiobjective approach for routing long-term complete area coverage mission with multiple heterogeneous UAVs, simultaneously considering the time to complete the mission and the number of recharging station to optimize the coverage tours; (2) locating the recharging stations that can be shared among the vehicles; (3) developing, evaluating, and comparing different heuristics that return the set of nondominated solutions to minimize the longest time among all the vehicles and the number of recharging stations.

The remainder of this work is organized as follows: In Section 2, a review of relevant related works is provided. A brief definition of the problem under investigation is provided in Section 3. A mixed-integer linear programming (MILP) formulation for the problem is presented in Section 4. The matheuristics to solve it are proposed in Section 5. Computational experiments to evaluate the proposed heuristics and a discussion on proposed strategies are presented in Section 6. Conclusions and final remarks are presented in Section 7.

## 2. Related Work

Coverage path planning (CPP) has been addressed by several studies, usually for robot applications in monitoring/surveillance missions that require the coverage of some determined target points in the environment. In particular cases, these monitoring missions must persist for a long time, following a path that maximizes each target’s visit frequency. Persistence is usually solved by implementing speed controllers for the robots to stay perpetually monitoring the environment, as proposed in [15]. The authors formulated a linear program (LP) that provides a solution for speed control in a dynamic environment. The robot path is planned a priori, and the proposed controllers adjust its speed to handle different levels of attention in different parts of the environment, keeping the coverage frequency bounded. Cassandras et al. [16] presented an optimal controller for a multiagent system in persistent monitoring. The focus was to control the multiple agents movements to minimize the uncertainty on the mission space. A deterministic finite-state formulation for this problem is presented in [17]. The authors proposed a branch and bound algorithm that quantifies the coverage quality in a finite prediction horizon.

A mixed-integer linear program (MILP) to find the fastest trajectory to cover a preassigned set of waypoints considering the collision avoidance constraints for UAV is presented in [18]. This solution was extended [19] by combining task assignment and trajectory planning for a fleet of UAVs, minimizing the mission time considering heterogeneous UAVs, different types of waypoints, time, and space constraints. Gonçalves et al. [20] and Keller et al. [21] explored the problem of collision avoidance for intersecting paths. The work in [20] was further extended in [22] to consider minimum spatial separation, acceleration constraints, and uncertainty in the speeds and positions of the vehicles.

Dille and Singh [23] proposed a heuristic for planning road coverage trajectory in sparse environments considering motion-constrained vehicles such as fixed-wing UAVs. In this case, road monitoring using an exhaustive sweeping movement is not considered the most appropriate. Thus, the authors converted the edge-oriented road graph into a node-graph representation (roads as nodes) and then built the solution based on a traveling salesman problem (TSP), ensuring the complete coverage of the roads by visiting all nodes. Unlike the previous node-based solution, Oh et al. [24] approached road patrolling with multiple fixed-wing UAVs considering it as an arc routing problem. An MILP formulation and an approximation algorithm based on the modified Dubins Chinese postman problem (mDCPP) as strategies to minimize the flight time were proposed.

Guerriero et al. [25] presented a multiobjective approach to the dynamic routing problem using UAVs. This strategy combines the task scheduling problem with the motion planning problem in a multicriteria optimization model. Their objectives considered the minimization of the distance traveled, the maximization of the customers visited, and the minimization of the number of UAVs, ensuring both spatial and temporal coverage of specific targets in the environment.

An important issue in practice that emerged from monitoring missions is the limited capacity of the vehicles. In this case, long-term coverage applications must consider the vehicle’s capacity constraints and possible tour interruptions for replenishment. As observed from the literature, several related works have investigated routing strategies using multiple depots to replenish the vehicles in logistic operations [26,27,28,29,30,31]. Another class of approaches simultaneously considers the routing and depot-locating problems. Generally, the objectives are defining the locations of the depots and assigning each one to a route so that path costs and the number of depots are minimized [32,33,34,35].

A particular case of this locating problem considers the depots as recharging stations due to the vehicle fuel constraint. This approach is usually applied for routing electric vehicles (EVs), which may require regular recharging, which can be a potential issue for EV applications. In this sense, Chung et al. [36] presented an optimization model for planning new charge station facilities over time, identifying predefined sites that maximize the total coverage flow. An approach for ranking these sites considering a multicriteria evaluation process integrated with a multiobjective method was proposed [37]. Riemann et al. [38] addressed the location of wireless charging facilities for roadway electrification. In this case, the EV equipped adequately with this technology can be recharged when driven over this type of road, also known as charging lanes. The authors formulated a mathematical model to locate a specific number of wireless charging lanes that maximizes the road traffic flow. Chen et al. [34] investigated the optimal deployment of the charging infrastructure that combines the static charging stations and the wireless charging lanes. The charging planning for autonomous vehicle logistic system was combined with the scheduling problem [39]. Ammous et al. [40] investigated the customers trip delay due to the charging time for an EVs allocation system, presenting a routing scheme that considers the charging station queues to minimize the expected overall trip time for all customers’ overall routes relative to their trip time, not considering the delay required for in-route charging.

For UAVs with VTOL capability, a different charging station technology was proposed [41,42], enabling vehicles to autonomously dock, recharge, and return to their missions. Another more advanced alternative is the station technology that swaps the battery, replacing the depleted battery with one fully charged [43]. It is not required to wait for the battery recharging that usually takes more time than the UAV’s flight time. As the most common UAVs have short endurance, determined by their battery capacity, such technologies have been considered in several solutions, especially for persistent missions.

The strategic deployment of such stations has been explored for package delivery services [44,45,46]. Hong et al. [44] proposed solutions to locate a limited number of recharging stations to maximize the customer’s coverage demand. Huang and Savkin [45] focused on minimizing the number of charging stations keeping the customer demand ratio and the connectivity among stations and the depot. Cokyasar [46] presented a mixed-integer nonlinear programming (MINLP) model that minimizes long-term investment and operational cost by defining delivery routes to customers using UAVs, trucks, or both vehicles (not for the same order). For the UAVs delivery, the charging stations are optimally localized to minimize their quantity, considering the UAV’s congestion costs at recharging stations and limited supply of batteries to be swapped. Due to the UAV payload restriction, such delivery works are constrained by one UAV delivering only one package to a customer at a time. The vehicle departs from the origin (warehouse) with the package and uses the charging structure to reach one target destination (customer), performing the same path to return to the origin. Thus, although some strategies for delivery can also be used in the context of surveillance, it is not straightforward to apply them in the problem considered in this work. In this paper, we propose to address the charging station locating problem jointly with the heterogeneous UAVs routing problem in a scenario that requires reaching more than one target in each UAV tour.

The routing considering the UAVs energy constraint was approached in some works as the fuel-constrained UAV routing problem (FCURP). Sundar and Rathinam [47] presented an MILP formulation and an approximated algorithm for FCURP to find a path for the UAV that visits all targets and some recharging stations, when required to satisfy the fuel constraint, keeping the recharging demand minimal. Mitchell et al. [48] extended the previous fuel constraint MILP formulation to multiple heterogeneous vehicles providing solutions for the multirobot persistent coverage problem (MRPCP). Two arc-based and two node-based MILP formulations for the extended FCURP for multiple UAVs (FCMURP) were analyzed [49]. In this case, the authors observed that the arc-based formulations outperformed the node-based formulations in computing the optimal solution for any instance of the problem. Another MILP formulation for a similar fuel constraint problem was proposed [50]. In this case, the authors aimed to minimize the overall costs to inspect critical locations when considering installing battery supply stations. The uncertainties in the fuel consumption by the UAVs for the FCMURP were investigated [51] by modeling the UAVs’ fuel consumption as a two-stage stochastic program that minimizes, in the first stage, the travel distances for all vehicles and, in the second, the additional costs to recharge when the vehicle fuel is inadequate to complete the route defined by the first stage.

A more specific robotic mission in CPP is the complete coverage problem, which requires the decomposition of the area into cells that the robot must entirely visit. Galceran and Carreras [52] presented several decomposition strategies, and the majority was classified as approximate or exact cellular decomposition, following [53]. Choset’s taxonomy mainly differentiates between decomposition types by their representation level of the target area.

Approximate decomposition is used by grid-based methods to represent the area with a set of uniform cells. This grid representation is only an approximation of the real area shape. In this case, the coverage is completed by visiting all cells, but the coverage completeness depends on the grid resolution. Albeit simple, the grid-based representation is not scalable due to the exponential growth in memory usage [54]. It also demands an accurate localization to maintain the coherency of the grid map for the coverage task, which becomes more challenging to apply to large areas [55,56].

Exact cellular decomposition [57] splits the free space into nonoverlapping cells that exactly fit the space geometry when joined. A simple way to perform this space coverage is using a sequence of back-and-forth movement (zigzag) inside each cell. The sequence of the coverage among the cells can be solved by representing the decomposed space with an adjacency graph and then applying a graph search algorithm to determine the coverage order. The robot achieves complete area coverage by combining the navigation among the cells following the specified sequence and the zigzag movement inside each cell. Some works explored the direction of the zigzag movement to optimize the robot path, as seen in Li et al. [58], who explored the optimal coverage path in exact decomposition, finding the sweep direction that minimizes the UAV’s turns. The authors proved that for area coverage using UAVs, decreasing the number of turns increases the path’s efficiency in terms of length, duration, and fuel consumption. The sweep direction problem was also explored in [59], considering operations with multiple UAVs.

Avellar et al. [1] presented a solution based on the vehicle routing problem (VRP) to minimize the total mission time in a complete area coverage task using multiple fixed-wing UAVs. In this case, the exact cellular decomposition method was performed to define the set of coverage rows that minimizes the vehicle turns. An MILP formulation was presented to route a team of UAVs considering practical issues to deploy this kind of vehicle, such as the time needed to prepare and launch them manually (setup time). In this case, the authors noted that the setup time has a relevant effect on the number of UAVs planned for the mission, especially when the number of human operators is smaller than the number of UAVs to be launched.

Similar area-coverage approaches using UAVs are reported in [8]. However, different from [1], the authors used the approximated cellular decomposition, presenting an MILP formulation to define the UAVs routes over the grid. This strategy of representing the area by a grid was also used in [9,60,61], which, despite being designed for area coverage applications, addresses different problems.

Li and colleagues [9,60] combined the environmental, fuel constraints, and charging station location problem for persistent area surveillance with multiple UAVs. Given the number of working vehicles and the number of charging stations to be opened, a genetic algorithm was proposed to optimize the open paths for each UAV and the recharging station placement to minimize the energy consumption and the flight time to visit some high-priority cells.

Acevedo et al. [61], albeit not considering the recharging of the UAV out of its base, proposed a distributed method for area division performed online among the UAVs. Each vehicle is assigned to a cell whose size is adjusted as the vehicles share information with their neighbors. All UAVs perform a planned closed path inside its cells that maximizes the periodic communications among the UAVs. As the information is updated, the cell dimension is adjusted to optimize the inside path to maximize coverage frequency considering the vehicles’ capacities.

Although some studies focused on the route optimization problems for multiple UAVs in the complete area in terms of coverage operations and others locating the recharging stations to extend the vehicle range in the mission, none of the above works considered all the constraints together in a multiobjective approach in a heterogeneous fleet. To the best of our knowledge, this work, for the first time, presents a multiobjective solution for routing long-term missions for complete area coverage with multiple heterogeneous UAVs, simultaneously considering the minimization of the total time of the mission and the localization of the minimum number of recharging stations. Note that the minimization of the number of recharge stations is a relevant criterion due to the costs associated with these facilities. However, the objectives may be conflicting, as reducing the number of recharging stations may force the UAV to travel a longer path to recharge, thereby increasing the time to complete the mission.

In our work, we are dealing specifically with a locating-route problem to guarantee complete area coverage by UAVs, which is not only restricted to area-monitoring tasks. The proposed methodology can be incorporated into the solution to other problems, such as using UAVs to supply data communication in wireless sensor networks (WSNs). The UAVs can also be used for data collection to support the transmission of the data gathered by sensor nodes on the ground. In this case, the vehicle capacity to transmit data is limited by the power consumption in the data transmission, flight speed, the available energy on the UAVs, the deployment location, and various other factors investigated in the literature, as presented in [62,63,64,65].

## 3. Problem Definition

Given an area to be monitored, it must be decomposed in several coverage rows considering sweep direction, camera parameters, and the UAV flight altitude [1]. The distance between any two rows is calculated as a function of the camera footprint width, determined by the UAV flight altitude, the image sensor width, the camera focal length, and the side overlap percentage. These parameters influence the captured image, and their impact in the image reconstruction was previously assessed [66].

The area is covered by a team of *k* UAVs equipped with on-board cameras pointing downward. The UAV flies at different heights to avoid collisions, which is compensated by adjusting the camera focus to keep the same footprint width for all vehicles. The UAVs have limited endurance and can reach at least one recharging station along their paths. They can recharge as many times as needed and must be able to cover at least one coverage row.

The decomposed area is modeled by a graph G=(V,E), where V=T∪D is the set of vertices, *E* is the set of edges, and N=|V| is the number of vertices of the graph. Let $T=\{t_{1},t_{2},\ldots ,t_{n}\}$ be the set of targets in the extremities of the coverage rows and $D=\{d_{0},d_{1},d_{2},\ldots ,d_{n}\}$ be the set given by the union of the depot and the potential locations for recharging stations $\left\{d_{1},d_{2},\ldots ,d_{n}\right\}$. The base station (depot) $d_{0}$ denotes the vertex where the UAVs start and end their routes. All edges (i,j)∈E have a non-negative value $\gamma_{i,j}$ and we consider the Euclidean distance between the vertices *i* and *j*. The distances are time-invariant, symmetric ($\gamma_{i,j}=\gamma_{j,i}$), and satisfy the triangle inequality, i.e., $\gamma_{p,q}+\gamma_{q,r}\geq \gamma_{p,r},\forall p,q,r\in V$. The coverage rows, $L_{all}\subseteq E$, are required edges defined by the decomposition method and must be covered once by some UAVs. The vehicle endurance is denoted by $F_{k}$, quantified by total flight time at a constant speed of $v_{k}$. The flight time, $F_{k}$, is reduced proportionally to the edge length given by $c_{i,j}^{k}=\gamma_{i,j}/v_{k}$. The time spent on the recharging station is considered proportional to the flight time, and the proportionality constant $q_{k}$ denotes its ratio.

The impact of UAV altitude on the coverage row generation relies on our group’s previous development [1]. The UAV’s low-level parameters are reflected in our model by the high-level input parameters, such as the UAV flight time ($F_{k}$), speed ($v_{k}$), the recharge time ratio ($q_{k}$), and the costs to traverse the edges ($c_{i,j}^{k}$). Aspects concerning different altitudes among vertices or extra costs to perform curves by specific vehicle models can be embedded in the $c_{i,j}^{k}$ parameter. For instance, we can consider the length of the real path that must be followed by a fixed-wing UAV to account for curvature constraints instead of using the Euclidean distance between the nodes. Other particularities can be mapped to their recharging time ratio $q_{k}$.

The objective of this work was to find the UAVs’ routes and locate the necessary recharging stations considering each vehicle’s performance, regardless of its hardware. Although the proposed method works independent of the UAV model, using either electric or combustion engines, in the remaining text, we consider the use of electric vehicles that complete their battery charge in the recharging stations available in the area.

**Figure 1:**
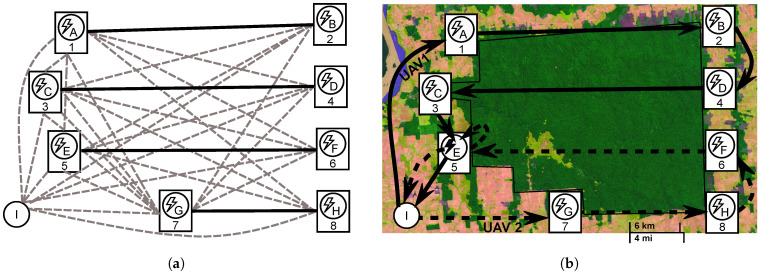
Example of the graph used to represent a coverage area and the solution illustrated in a preservation area image. (**a**) The recharging stations that can be opened in the area are represented by A–H, the depot is the vertex (I), and the targets are identified by the numbers 1–8. (**b**) An illustration of coverage routes applied to preservation area monitoring. Deforestation map of the Amazon from the TerraBrasilis website [68].

Figure 1 illustrates a graph and the solution for area monitoring. The complete graph in Figure 1a contains edges to connect every pair of different vertices in the set *V*. The coverage rows $L_{all}$ are all the continuous lines. Their ends (targets) must be numbered, using odd numbers on one side of the area and even numbers on the other. The recharging stations are aligned with the targets, being identified in the figure by letters (A–H) and the UAVs depot (I). Figure 1b depicts an example of monitoring a protection area [67]. In Brazil, this region has several hundred demarcated indigenous lands, which are essential for the protection of indigenous peoples. Thus, as illustrated, the active monitoring of these areas is crucial. The solution shows two routes for different UAV models that require recharging to complete the coverage mission. Due to the cost associated with installing new recharging stations in this location, more emphasis was placed on minimizing recharging stations opening to the detriment of time to complete the longest route. In this case, the solution that shares the recharging station at vertex *E* was considered the best one.

Next, in Section 4, the mathematical formulation is presented to find the exact solution for small areas and, in Section 5, matheuristic strategies are explained that quickly return feasible solutions to cover larger areas.

## 4. Mathematical Formulation

The mathematical formulation presented in this work was inspired by [1] for area coverage, by [49] for the arc-routing model, and by [48] for the heterogeneous vehicles application. Besides combining these different characteristics to minimize the longest route time, we also considered locating the recharging stations that can be open in the vertices defined using the area decomposition method while simultaneously minimizing the number of such stations.

The modeling of these two objectives is relevant. The first objective, which is to minimize the longest vehicle route ($f_{1}$), is crucial for monitoring or search-and-rescue operations that demand the accomplishment of the mission in the least time. The second objective ($f_{2}$), which is to minimize the number of recharging stations, is important for practical reasons as there might be financial costs associated with their installation, maintenance, and operation. The minimization of the number of stations is essential for some scenarios, as observed for fixed-wing UAVs, which might require a dedicated human operator responsible for the launch and rescue of the vehicle.

Let the decision variables be: $x_{i,j}^{k}\in \left\{0,1\right\}$ (1 if the *k*th UAV flies from vertex *i* to *j*); $y_{d}\in \left\{0,1\right\}$ to indicate whether or not the recharging station $d\in D\backslash \{d_{0}\}$ is used; $z_{i,j}^{k}\in R$ is a flow variable that denotes the total flight time performed by the UAV *k*, starting the timing at any vertex in *D*, which defines the time that the UAV reaches *j* after passing through vertex *i*; and $P_{max}\in R$ is the longest time to finish the planned route among all UAVs. The optimal coverage problem can be formulated as in (1)–(16), minimizing the mission time ($f_{1}$) and the openings of recharging stations ($f_{2}$) subject to the following constraints:

(1) $$\min\;f_{1}=P_{max}$$

(2) $$\min\;f_{2}=\sum_{d\in D}y_{d}$$

(3) $$\begin{array}{l} \mathrm{Subject}\;\mathrm{to}:\\ \sum_{i\in V}\sum_{j\in V}\left(1+q_{k}\right)x_{i,j}^{k}c_{i,j}^{k}\leq P_{max},\;\forall k\in K, \end{array}$$

(4) $$\sum_{i\in V}x_{i,j}^{k}=\sum_{i\in V}x_{j,i}^{k},\;\forall j\in V,k\in K,$$

(5) $$\sum_{k\in K}\sum_{i\in V}x_{i,j}^{k}=1,\;\forall j\in T,$$

(6) $$\sum_{i\in V}x_{d_{0},i}^{k}=1,\;\forall k\in K,$$

(7) $$y_{d}\leq \sum_{k\in K}\sum_{i\in V}x_{d,i}^{k}\leq Ny_{d},\;\forall d\in D\backslash \left\{d_{0}\right\},$$

(8) $$x\left(\delta^{+}\left(S_{u}^{k}\right)\right)\geq y_{d},\;\forall k\in K\;d\in S_{u}^{k}\cap D,\;S_{u}^{k}\subset V\backslash \left\{d_{0}\right\},$$

(9) $$\sum_{j\in V}z_{i,j}^{k}-\sum_{j\in V}z_{j,i}^{k}=\sum_{j\in V}c_{i,j}^{k}x_{i,j}^{k},\;\forall i\in T,\;k\in K,$$

(10) $$z_{d,i}^{k}=c_{d,i}^{k}x_{d,i}^{k},\;\forall i\in V,\;d\in D,\;k\in K,$$

(11) $$z_{i,j}^{k}\leq \left(F_{k}-t_{j}^{k}\right)x_{i,j}^{k},\;\forall k\in K,\;j\in T,\left(i,j\right)\in E,$$

(12) $$z_{i,d}^{k}\leq F_{k}x_{i,d}^{k},\;\forall i\in V,\;d\in D,\;k\in K,$$

(13) $$z_{i,j}^{k}\geq \left(s_{i}^{k}+c_{i,j}^{k}\right)x_{i,j}^{k},\;\forall k\in K,\;i\in T,\left(i,j\right)\in E,$$

(14) $$\sum_{k\in K}x_{i,i+1}^{k}+\sum_{k\in K}x_{i+1,i}^{k}=1,\;\forall i\in T\backslash \left\{2,4,6,\ldots \right\},$$

(15) $$x_{i,j}^{k}\in \left\{0,1\right\},\;\forall k\in K,\;\left(i,j\right)\in E,$$

(16) $$y_{d}\in \left\{0,1\right\},\;\forall d\in D\backslash \left\{d_{0}\right\}.$$

The objective in (1) is to minimize the longest time among the UAVs, $P_{max}$. This is a min-max problem where the decision variable $P_{max}$ is an upper bound on the UAV’s time to cover the edges ($x_{i,j}^{k}c_{i,j}^{k}$) plus its recharging time given by ($q_{k}x_{i,j}^{k}c_{i,j}^{k}$) in constraint (3), where $q_{k}$ is the ratio of time for the UAV *k* to recharge given the time consumed to traverse the edge. The second objective $f_{2}$ is to minimize the total sum of recharging stations, each one represented by $y_{d}$, as stated in (2).

The degree constraint in (4) controls the flow through the vertices, ensuring whenever a UAV *k* arrives at the vertex, it must leave it. Constraint (5) guarantees that each target is visited by only one UAV. To enforce all UAVs leaving the base $d_{0}$, the constraint (6) is used.

The constraint (7) guarantees the open status of the recharging stations $y_{d}=1$ if any UAV departs from it ($x_{di}^{k}=1$); otherwise, it is kept closed $y_{d}=0$. An open station $y_{d}$ can be connected with at least one and at most *N* vertices (total number of vertices in the graph).

Constraint (8) prevents subtours when recharge is required, keeping the solution connected. Let, for any UAV k∈K, the subsets $S_{u}^{k},S_{v}^{k}\subset V,\;\delta^{+}\left(S_{u}^{k}\right)=\left\{\left(i,j\right)\in E:i\in S_{u}^{k},\;j\in S_{v}^{k},\;S_{u}^{k}\cap S_{v}^{k}=\emptyset \right\}$ and $x\left(A\right)=\sum_{i,j\in A}x_{i,j}^{k},\;\forall A\subseteq E$. This constraint eliminates the subtours that can occur when some UAVs visit a recharging station. The subset of vertices $S_{u}^{k}$ represents any subtours without the base $d_{0}$, and the constraint (8) ensures at least one departure of UAV *k* from a vertex in $S_{u}^{k}$ to any vertex in other subtours represented by $S_{v}^{k}$. The high number of restrictions can make the computational effort inefficient if we add all the necessary restrictions during the MILP model’s construction. In this case, a possible solution for this issue is to relax the constraint from the formulation, and whenever a feasible integer solution is obtained, a callback method checks if it is violated. This violation is identified if the solution has more than one strongly connected component for any UAV, which means the occurrence of subtours in the vehicle route. All violated constraints are then added to the model, and the optimization of the original problem continues. More details about this method are provided in [49].

The constraint (9) defines the flow variables $z_{i,j}^{k}$, setting the time that the UAV *k* visits a vertex *j* from the target *i*. This restriction guarantees the order of visits, preventing subtours over the targets. The constraints (10)–(13) guarantee that the tour time performed by a vehicle until reaching any vertex in *D* (recharging station or depot) does not exceed its flight capability, $F_{k}$. In this case, constraint (10) defines the flow value after the vehicle leaves the recharging stations, setting the time to visit the vertex *i*. As the tour timing starts from a vertex in *D*, the flow value is defined by the time the UAV spends to traverse the edge given by $c_{d,i}^{k}$.

As $F_{k}$ defines the maximum flight time of vehicle *k*, the flow value $z_{i,j}^{k}$ to visit the target *j* must guarantee a sufficient battery charge (flight time) to visit at least one vertex in *D* after visiting the target *j*. In this case, the constraint (11) upper bounds the $z_{i,j}^{k}$, as the UAV should have at least the minimal battery charge on target *j* to reach its nearest vertex in *D*, given by $t_{j}^{k}$ ($t_{j}^{k}=\min_{d\in D}c_{jd}$). The constraint (12) upper bounds the flow value to visit a vertex in *D* from any vertex, as the UAV can only spend its maximum flight time $F_{k}$ to traverse a single edge if both vertices are in *D*. The constraint (13) lower bounds the flow value to the minimum flight time required for the vehicle to reach the vertex *j*, considering the minimal time required to arrive at target *i*. This is defined by the flight time from the nearest vertex of *i* in *D*, given by $s_{i}^{k}$ ($s_{i}^{k}=\min_{d\in D}c_{di}$).

Constraint (14) enforces the coverage row task. This is achieved by ordering target visits given its numerical identification. Lastly, the constraints (15) and (16) impose the decision variables $x_{i,j}^{k}$ and $y_{d}^{k}$ as binary restrictions.

## 5. Solution Strategies

As the computational cost to solve large instances with the exact method is restrictive for practical applications, it is acceptable to use some heuristic strategies to efficiently provide satisfactory solutions. Thus, this section presents four matheuristic approaches based on MOVNS [69], since a similar metaheuristic was demonstrated to be effective for solving vehicle routing problems [70].

The proposed matheuristics divide the original problem into smaller subproblems that are easier to solve, and the exact method is used, depending on the strategy, to find routes on subgraphs (clusters) or improve a solution. Here, the coverage rows, UAVs, and the recharging stations are grouped in clusters, which should be distributed appropriately, considering both objectives of this work.

The MOVNS works on the clustering problem, exploring the search space by making systematic changes in the clusters. These changes are performed by the proposed neighborhood functions, generating different routes by changing the distribution of coverage rows, UAV models, and the recharging stations.

The different matheuristics were designed to check the effect over the quality and time to compute the returned solutions. In the first approach, the neighborhood functions perform simple movements like changing coverage rows and UAV models among routes or shutting down some recharging stations. After that, the exact method is called for routing every changed cluster. As this strategy is characterized by the intensive use of the exact method, other approaches were designed to investigate the effect of different neighborhood functions that also use heuristics for routing to mitigate the exact method calls. Such calls were significantly decreased in the second and third approaches and reduced to only once in the last one. In the next sections, we provide the details of the proposed approaches.

### 5.1. Solution Representation

A candidate solution is represented by the set of routes $X=\{x_{1},x_{2},\ldots ,x_{k}\}$ for a fleet of *k* UAVs, where $x_{i}=\langle v_{1},v_{2},\ldots ,v_{|x_{i}|}\rangle$ is a sequence of vertices and $v_{|x_{i}|}\in V$. The set of nondominated solutions is represented by $\mathrm{Front}=\{X_{0},X_{1},\ldots ,X_{\left|\mathrm{Front}\right|}\}$. All information necessary to compute the route $x_{i}$ is encoded in a data structure we denote as a cluster. The cluster *i* is composed of the subset of coverage rows $L\left(x_{i}\right)=\left\{l_{1},l_{2},\ldots ,l_{m}\right\}$, the UAV model identifier $m(x_{i})$, and $S(x_{i})$, the set of recharging stations available in the cluster.

The vertices where the recharging stations can be installed are represented by $d_{1},\ldots ,d_{\left|D\right|}$, and the UAVs base station (depot) is identified by $d_{0}$. All opened recharging stations for the solution X is represented by $S_{all}={⋃}_{i=1}^{k}S\left(x_{i}\right)$, and the set of required edges called coverage rows is represented by $L_{all}={⋃}_{i=1}^{k}L\left(x_{i}\right)$.

### 5.2. Multiobjective Variable Neighborhood Search (MOVNS)

The MOVNS is a metaheuristic that returns the set of nondominated solutions exploring the neighborhood changes systematically. In this case, the variable neighborhood descent (VND) is performed as a local search with perturbation to escape from local minimum [71]. The general steps of the proposed metaheuristics are described in Algorithm 1.
**Algorithm 1:** Multiobjective variable neighborhood search (MOVNS).
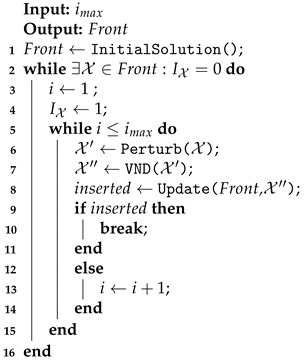


The MOVNS receives the maximum number of iterations ($i_{max}$) as input and returns the nondominated solutions on the front set. After the initialization (line 1), the VNS is performed for any nondominated solution on the front set for which the neighborhood was not investigated, such that $I_{X}=0$ (lines 2–16). In each iteration, the solution is perturbed, and the VND is performed. The perturbation (line 6) randomly operates in both processes: the openings of recharging stations and the coverage rows shift among the routes. After the current solution is perturbed, the VND is performed to find other nondominated solutions (line 7). All solutions found by VND are evaluated, and the front is updated considering the Pareto dominance relation (line 8). For any feasible solution X found by the VND, the domination relationship evaluates both objectives: $f_{1}\left(X\right)$, the cost of the longest flight route; and $f_{2}\left(X\right)$, the number of recharging station used in the route. In this case, it is considered that X dominates another solution $X^{\prime }$ if ∃i∈{1,2}, such that $f_{i}\left(X\right)<f_{i}\left(X^{\prime }\right)$ and $f_{i}\left(X\right)\leq f_{i}\left(X^{\prime }\right),\forall i\in \left\{1,2\right\}$. If any nondominated solution is inserted on the front, then the inner loop is broken (line 10) and the VNS is restarted considering another random solution in the front that has not yet been investigated. More details are provided below.

#### 5.2.1. Initialization

**Figure 2:**
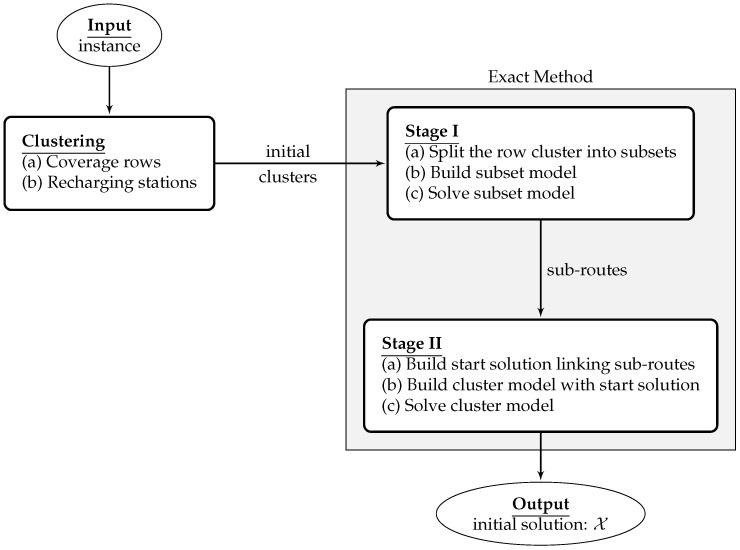
Scheme of the methods used in initialization.

More generally, the initial solution is built by the clustering process, splitting the original problem into clusters and then applying the exact method to find routes in each one of such clusters. In Figure 2, the scheme to generate the initial solution is presented. First, the clustering procedures are applied to assign the UAVs, the coverage rows, and the recharging stations to the clusters, and then a two-stage exact method is applied in the routing phase.

##### A. Clustering

The first step of the initialization is clustering, which assigns the coverage rows to the UAVs considering their performance. The row set of all coverage rows $L_{all}$ is split into subsets given the UAV flight range, the distances between rows, and their lengths. After defining the coverage row subset to every vehicle, the recharging stations they can visit are identified and assigned to the recharging station subsets. These subsets and the vehicle identifier compose a cluster, grouping all data required to build the UAV route.

**Figure 3:**
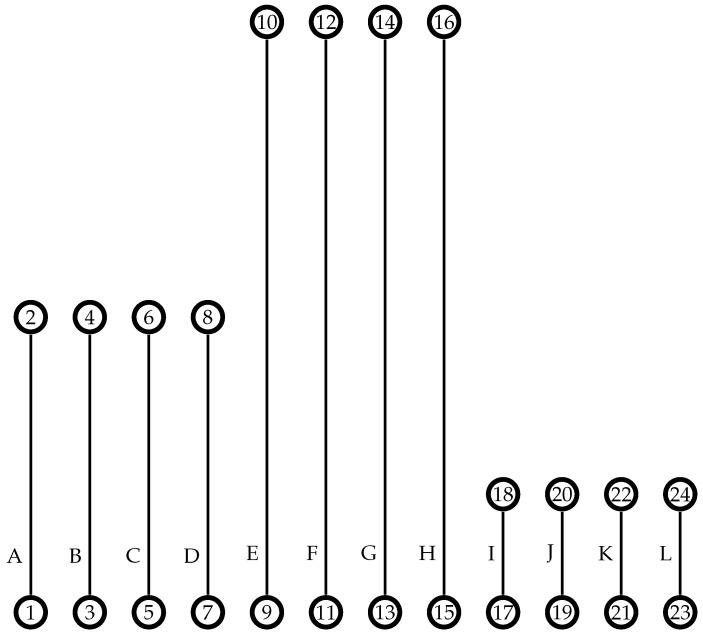
Example of instance used to illustrate the initialization of the MOVNS.

To illustrate the initialization procedures as they are presented, Figure 3 is used to exemplify the initial solution generation. Let the length of rows be A=B=C=D=5 km, E=F=G=H=10 km, I=J=K=L=2.5 km, and the distance between the rows calculated by the area decomposition method [1] is 0.5 km. Consider that two heterogeneous UAVs are available to perform this coverage task. The vehicle in cluster $x_{1}$ has $q(m\left(x_{1}\right))=1$, which means that it requires the same amount of its flight time capability, $F_{1}$, to completely charge the battery. The other vehicle in $x_{2}$ has lower performance, with a ratio $q(m\left(x_{2}\right))=2$, which means it requires twice its flight time to charge its battery at a recharging station.

The coverage rows assignment procedure is described in Algorithm 2 using the instance problem shown in Figure 3 as an example of input. Initially, the total coverage length, calculated as the sum of all row lengths, is divided among the vehicles considering their performance (lines 1–7). A portion of the total coverage length is defined for each UAV, $r_{x_{i}}$, limiting the assignment of rows with regards to their lengths. The variable $r_{x_{i}}$ is not bounded by the vehicle’s real flight capacity, $F_{x}$: this variable is only an estimate used to balance the coverage row distribution, considering the row length and the relative vehicle performance. The $r_{x_{i}}$ of each UAV is based on the recharging-flight time ratio $q(m\left(x_{i}\right))$, assigning more coverage area to the vehicle with the lowest q-value (i.e., its coverage area must be greater than that of other vehicles with a higher q-value). For this, the vehicle’s performance is evaluated based on the q-value of the best model (line 3). Then, the amount of coverage for each UAV is estimated by splitting the total coverage length according to the ratio between the vehicle performance and the overall performance $p_{x_{i}}÷p_{all}$ (line 7). In the example, the sum of all row lengths in $L_{all}$ (total_length) is 70 km, $q_{min}=1$, resulting in $r_{x_{1}}=0.67\times 70=46.9$ km and $r_{x_{2}}=0.33\times 70=23.1$ km.

After, the rows are sorted from the longest to the shortest (line 8), resulting in the sequence 〈E,F,G,H,A,B,C,D,I,J,K,L〉. The estimated coverage length assigned to the vehicle is used to sort the clusters in descending order (line 9). In the example, this step keeps the order as $X=\langle x_{1},x_{2}\rangle$. Next, each UAV row set is assigned a row considering the row length order and the vehicle performance (line 11). In the example, the first rows assigned are $L\left(x_{1}\right)=\left\{E\right\}$ and $L\left(x_{2}\right)=\left\{F\right\}$. After, $r_{x_{i}}$ is updated (line 12), making $r_{x_{1}}=46.9-10=36.9$ km and $r_{x_{2}}=23.1-10=13.1$ km. Then, $l_{i}$ is removed from the set $L_{all}$ (line 13), finishing these iterations with $L_{all}=\langle G,H,A,B,C,D,I,J,K,L\rangle$.

Next, each cluster receives the nearest rows from the one previously assigned, while the sum of their lengths does not exceed the current $r_{x_{i}}$ (lines 15–26). The proximity between coverage rows is measured by the distance from row *l* to every $l_{u}\in L_{all}$. Since the rows can have different lengths, the proximity is calculated as the sum of the distances between their targets (line 17). Given two rows (*l*,$l_{u}$), this distance function returns the least sum of both edges lengths connecting (*l*–$l_{u}$) by their vertices. For example, the proximity of the (E,I) rows is the sum of the lengths of edge (9, 17) and the edge (10, 18). After calculating the proximity of rows represented by $\gamma_{l,l_{u}}$, this measure is used to sort the coverage rows (line 18). This sorting phase, in the example, as the first row assigned to the cluster $x_{1}$ is *E*, returns the sequence 〈G,H,D,C,B,A,I,J,K,L〉. Afterward, the coverage row $l_{u}$ is assigned to the cluster $L(x_{i})$, following the distance order in $L_{all}$, while the row length does not exceed the UAV current $r_{x_{i}}$ (lines 19–25). At the end of these steps, the distribution of the rows in the instance is $L\left(x_{1}\right)=\left\{B,C,D,E,G,H\right\}$ and $L\left(x_{2}\right)=\left\{F,I,J,K,L\right\}$, with the row *A* in $L_{all}$ remaining. This row was not distributed as its length, 5 km, exceeds the length control variable of both vehicles $r_{x_{1}}=1.9$ and $r_{x_{2}}=3.1$ km.
**Algorithm 2:** Coverage rows assignment.
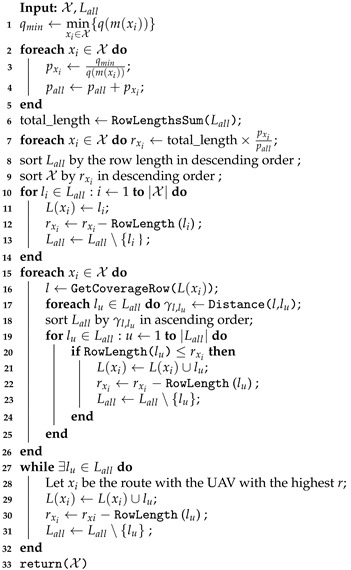


The remaining rows are incrementally assigned to the clusters with the highest *r* while the set $L_{all}$ is not empty (lines 27–32). In the example, as $r_{x_{2}}$ has the highest r-value, the coverage row (*A*) is assigned to $L(x_{2})$. Thus, the assignment procedure returns the clusters with $L\left(x_{1}\right)=\left\{B,C,D,E,G,H\right\}$ and $L\left(x_{2}\right)=\left\{A,F,I,J,K,L\right\}$.

The last procedure of the clustering phase is to assign a recharging station to every target’s location. Given the set of clusters X, the recharging station procedure must be able to open a set of stations denoted by $S(x_{i})$ in all targets of $L(x_{i})$. In this case, we add a new vertex in the graph in the same location as the target vertices. After, these vertices are assigned to the recharging station set of each UAV. This procedure adds an extra vertex to each target in Figure 3, resulting in the sets $S\left(x_{1}\right)=\left\{3^{\prime },4^{\prime },5,6^{\prime },7^{\prime },8^{\prime },9^{\prime },10^{\prime },13^{\prime },14^{\prime },15^{\prime },16^{\prime }\right\}$ and $S\left(x_{2}\right)=\left\{1^{\prime },2^{\prime },11^{\prime },12^{\prime },17^{\prime },18^{\prime },19^{\prime },20^{\prime },21^{\prime },22^{\prime },23^{\prime },24^{\prime }\right\}$.

##### B. Routing

The route is calculated by a two-stage approach aiming to speed up the optimization process with a start solution. In the first stage, each row set $L\left(x_{i}\right)$ in the cluster $x_{i}$ is split into smaller row subsets $L\left(x_{i}^{1}\right),L\left(x_{i}^{2}\right),\ldots ,L\left(x_{i}^{v}\right)$ with a defined maximum number of rows. Next, each model subset is solved by the exact method at Stage I. The subroutes are then linked to create a start route used as a warm-start in exact method of Stage II. This start solution may not be optimal, but it can offer a suitable starting point for the second stage, reducing the computation time.

**Figure 4:**
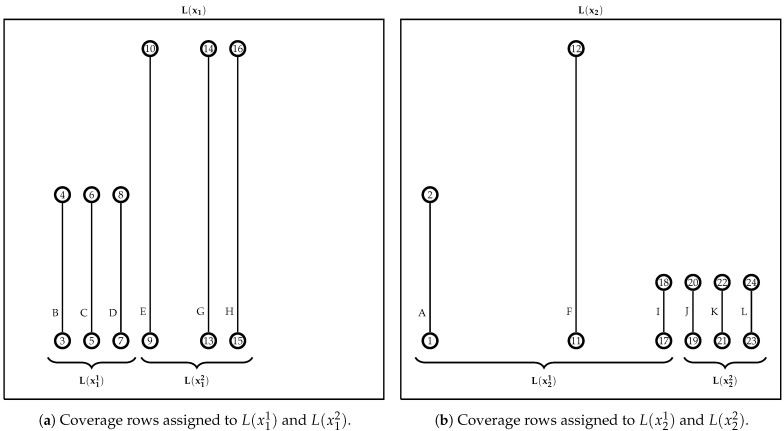
Splitting $L(x_{1})$ and $L(x_{2})$ into their respective subclusters $L(x_{1}^{1})$, $L(x_{1}^{2})$, $L(x_{2}^{1})$, and $L(x_{2}^{2})$.

Given the row set of each cluster $L(x_{i})$, determined by Algorithm 2, and the maximum number of coverage rows into the subset $l_{max}$ (a user-defined parameter), the first step of the splitting procedure is defining the number of row subsets $n_{i}$ for each $L(x_{i})$ to balance its row number. Let $l_{max}=5$ for the example shown in Figure 4. Each UAV was assigned six coverage rows $|L\left(x_{1}\right)|=|L\left(x_{2}\right)|=6$, which exceeds $l_{max}=5$. In this case, the splitting method divides the row set $L(x_{1})$ into $L(x_{1}^{1})$ and $L(x_{1}^{2})$, both with the same number of coverage rows ($w_{1}^{1}=w_{1}^{2}=3$), and $L(x_{2})$ was split into $L(x_{2}^{1})$ and $L(x_{2}^{2})$ with $w_{2}^{1}=w_{2}^{2}=3$. This coverage row distribution must define the number of subsets $n_{u}$ needed to keep as many coverage rows as possible and with a balanced number of rows among them ($w_{i}^{u}$). This procedure, for the example, avoids non balanced subclusters like $w_{1}^{1}=5$ and $w_{1}^{2}=1$ or adding a nonrequired subcluster resulting in $w_{1}^{1}=2$, $w_{1}^{2}=2$ and $w_{1}^{3}=2$.

The number of the row subsets ($n_{i}$) and their quantity of rows ($w_{i}^{u}$) are set up in the initialization of Algorithm 3 (line 1). Next, the coverage row set $L(x_{i})$ is divided into $n_{i}$ subsets, and each subset $u=\{1,\ldots ,n_{i}\}$ is assigned at most $w_{i}^{u}$ coverage rows, considering the vertex positions and the proximity among them (lines 2–17). The rows in $L(x_{i}^{\prime })$ are sorted from the leftmost row to the rightmost (line 5). In the example shown in Figure 4b, the sorting for splitting $L(x_{2})$ returns the sequence 〈A,F,I,J,K,L〉. Afterward, the first row of this sequence is assigned to the current subset $L(x_{i}^{u})$ (line 7). This step for $L(x_{2})$ results in $L\left(x_{2}^{1}\right)=\left\{A\right\}$.
**Algorithm 3:** Splitting into subclusters.
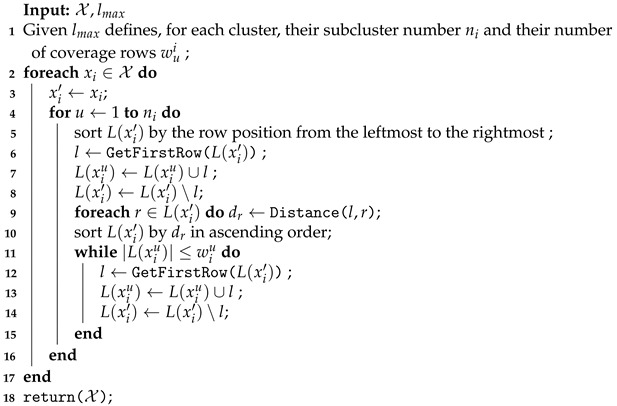


The distances from the row *l* to the others on set $L(x_{i}^{\prime })$ are calculated (line 9) using the same distance function described in Algorithm 2. The rows in $L(x_{i}^{\prime })$ are sorted by their proximity measure $d_{r}$ in ascending order (line 10). In the example, the sort method returns the sequence 〈F,I,J,K,L〉. Even with the coverage row *F* being the longest row, it is considered nearer to *A* than *I*, $d_{F}<d_{I}$ (i.e., Distance(A,F)<Distance(A,I)). The nearest rows are assigned to the subset $L(x_{i}^{u})$ and removed from the set $L(x_{i}^{\prime })$ (lines 11–15). After running this internal *while* loop to the example of the coverage rows, the subset assignment results in $L\left(x_{2}^{1}\right)=\left\{A,F,I\right\}$. Then, the process continues to the next coverage row set, $L(x_{2}^{2})$. In the end, $L(x_{1})$ is split into $L\left(x_{1}^{1}\right)=\left\{B,C,D\right\}$ and $L\left(x_{1}^{2}\right)=\left\{E,G,H\right\}$, as shown in Figure 4a; the rows in $L(x_{2})$ are divided into $L\left(x_{2}^{1}\right)=\left\{A,F,I\right\}$ and $L\left(x_{2}^{2}\right)=\left\{J,K,L\right\}$ subsets, as shown in Figure 4b.

After defining all subsets, the routing is solved through an MILP model. The mathematical formulations used in both Stage I and Stage II are quite similar to the one presented in Section 4 but with small modifications to solve a mono-objective problem for one vehicle. In this case, the exact method is used only to minimize the route time.

**Figure 5:**
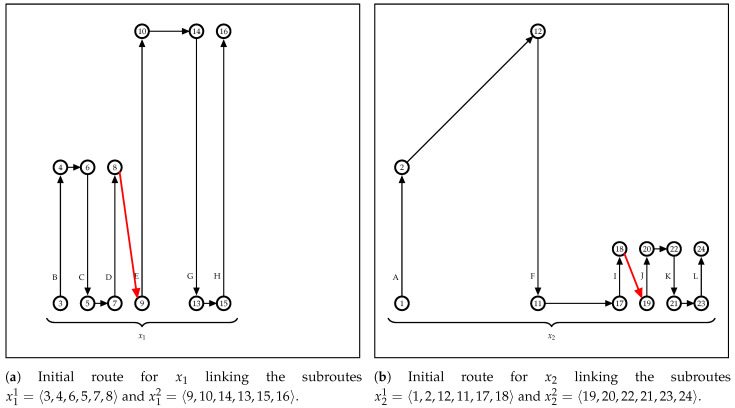
Example of subroutes linked to build the route $x_{1}$ and $x_{2}$.

The cluster subroutes computed in Stage I are linked by Algorithm 4, building an approximated route for each UAV. These routes are used as a starter solution to build the UAV optimal route in Stage II. Initially, the cluster *i* with the leftmost input vertex among its subroutes is selected (line 3). In the example shown in Figure 5a, this step checks the leftmost coverage row between *B* and *E*, which are the first coverage rows visited by the UAVs in their respective subroutes $x_{1}^{1}$ and $x_{1}^{2}$. The route $x_{i}$ is initialized with the previously selected subroute *c* (line 4). In the example, these steps assign to the current empty route $x_{1}$ the subroute $x_{1}^{1}$, which is represented by the vertices sequence $x_{1}=\langle 3,4,6,5,7,8\rangle$.

Next, the other subroutes in the cluster are selected and integrated into the route being built, following its proximity (lines 5–13). The subroute $x_{i}^{u}$ selection is performed by line 6, considering the distance between the output vertex on the last covered row in route $x_{i}$ and the first vertex visited by the vehicle on the remaining subroutes. This step for the Figure 5b example checks the distance from vertex 8, the $x_{1}$ output vertex, to vertex 9, which is the input vertex in the $x_{1}^{2}$ subroute. In this case, the battery charge of the UAV to depart from the output vertex in $x_{i}$ must be sufficient to reach the input vertex of the sub-route $x_{i}^{u}$ to link them directly (i.e., red rows in Figure 5) or by a path passing through recharging stations, if necessary. The function LinkSubRouteOnRoute evaluates the vehicle capacity and returns the appropriate links to connect the subroute $x_{i}^{u}$ in the route $x_{i}$. If this connection is infeasible, the built route is deleted, and the link process continues to the next cluster (lines 9–12). For the clusters with empty routes, the exact method in Stage II builds the MILP model without an initial starting point.
**Algorithm 4:** Linking subroutes to build a start solution.
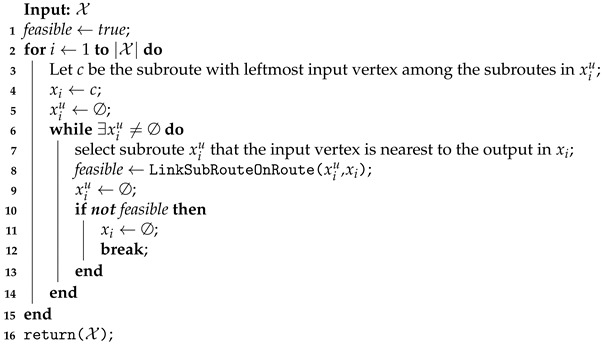


#### 5.2.2. Variable Neighborhood Descent (VND)

In the proposed MOVNS-based matheuristic, the VND is the method that iteratively alternates the functions developed to modify the current solution. These functions are denoted as neighborhood functions, and the solution generated by a neighborhood is called a neighbor. The neighborhood function is used to map the current solution to its neighbor, which is another feasible solution. If the neighbor satisfies the update rules, then it is accepted as a new incumbent solution [72].

**Figure 6:**
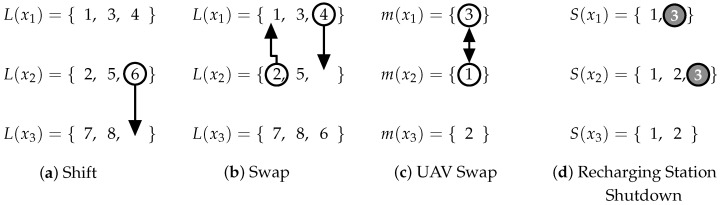
Illustration of actions executed by the neighborhood functions.

In this work, we implemented four neighborhood functions, as shown in Figure 6. The Shift function moves a coverage row $l\in L(x_{i})$ to other row set $L(x_{j})$, as shown in Figure 6a; the coverage row 6 from $L(x_{2})$ is moved to $L(x_{3})$. The Swap function exchanges rows among routes, reallocating a row $l_{1}\in L\left(x_{i}\right)$ to $L(x_{j})$ and a row $l_{2}\in L\left(x_{j}\right)$ to $L(x_{i})$. In Figure 6b, rows 2 and 4 change clusters. The row 2 from $L(x_{2})$ is transferred to $L(x_{1})$, and row 4 from $L(x_{1})$ is transferred to $L(x_{2})$. UAV Swap was designed to interchange the UAV models between two routes, transferring a vehicle of model $m(x_{i})$ from route $x_{i}$ to route $x_{j}$, while the current UAV of model $m(x_{j})$ assigned to $x_{j}$ is transferred to route $x_{i}$. In Figure 6c, the UAV of model 3 from route $x_{1}$ is transferred to the route $x_{2}$, and the UAV of the model 1 in $x_{2}$ is transferred to $x_{1}$. The Recharging Station Shutdown removes a recharging station from all routes that use this station. In Figure 6d, recharging station 3 is removed from routes $x_{1}$ and $x_{2}$.

The general structure of the VND is shown in Algorithm 5. Initially, the set of routes in the input solution X is assigned as the current best solution $X^{\prime }$ (line 1). After, the set of neighborhood functions N is defined, where $n_{1}$, $n_{2}$, $n_{3}$, and $n_{4}$ are the Shift, Swap, UAV Swap, and Recharging Station Shutdown functions, respectively (line 2).
**Algorithm 5:** Structure of the variable neighborhood descent (VND) search.
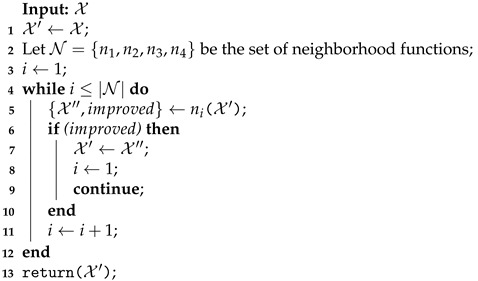


At any given iteration, a neighborhood function is applied to search for a better solution. If the function $n_{i}$ obtains any improvement, it returns the better solution $X^{\prime \prime }$ and the flag *improved* is set to *true*. Next, the current best solution is updated (line 7), the index *i* that controls the sequence of the neighborhood function is reset (line 8), and the VND iteration is broken (line 9) and continues with the next VND iteration applying the Shift function again. Otherwise, if the $n_{i}$ function obtains no improvement (*improved* = *false*), the index *i* is incremented (line 11), and in the next VND iteration, the next neighborhood function is applied. The iteration of VND stops when all neighborhood functions obtain no improvement.

#### 5.2.3. Perturbation

The perturbation designed for this work implements two functions: one explores the coverage row changes between routes, and the other function works to open recharging stations. These functions are randomly applied, with it being possible to apply one or both functions in any order for the last case.

The perturbation performed in the coverage row sets is based on the Shift neighborhood function represented in Figure 6a. In this case, any row from a random route is chosen and transferred to another route. The other perturbation function acts on the recharging-station-locating process by opening some stations. Unlike Figure 6d, this operation randomly chooses some shuttered stations to be opened. Next, the feasibility of the opening operation is checked. A recharging station is only opened if any route time is improved.

### 5.3. Matheuristics Approaches Based on MOVNS

The matheuristics proposed in this paper combine the MOVNS and mathematical programming methods to find high-quality solutions. In addition to using the MILP formulation to obtain the initial solution, the same two-stage method presented in the initial routing phase interoperates with the VND in different ways in the first three strategies presented in this subsection.

The designed modifications in the VND approach impact the matheuristic performance regarding the quality of the generated fronts and the computation time. Each matheuristic is classified according to the application of the exact method in the associated VND. First, the MOVNS with the exact method for routing (EMR) is presented, which uses the exact method to route all cluster changes. After, other approaches are proposed: the method that applies the exact method selectively (exact method applied selectively (EMAS)), the one in which the exact method acts as a neighborhood function (exact method as neighborhood function (EMNF)), and the method that does not use the MILP formulation in the VND (VND without exact method (WEM)). Table 1 summarizes the main differences among these approaches.

#### 5.3.1. Exact Method for Routing (EMR)

The EMR is a matheuristic characterized by solving the MILP model to route every change in a cluster performed by the neighborhood functions. Every new cluster designed by the row exchange, UAV model change, or shutting down of some recharging stations requires the exact method for routing. Next, we provide more details on the applied neighborhood functions.

The change in the coverage row sets made by the EMR Shift function is described by Algorithm 6. Initially, the longest route (highest time cost) $x_{i}$ is taken from the solution X (line 1), and the set $X^{\prime }$ that is modified by the row shift is initialized (line 2). Next, as long as there is no improvement in the solution and for any other route $x_{j}$ not yet augmented by the $x_{i}$ rows, a new set without *l* from $L(x_{i})$ is assigned to the route $x_{i}^{\prime }$ (line 5). The removed row is inserted in the set $L(x_{j}^{\prime })$ of $x_{j}^{\prime }$ (line 6).These new clusters are routed by the MILP function (lines 7–8); the new solution $X^{\prime }$ and the value true are returned if an improvement is found (lines 9–11).
**Algorithm 6:** Shift neighborhood function performed on exact method for routing (EMR).
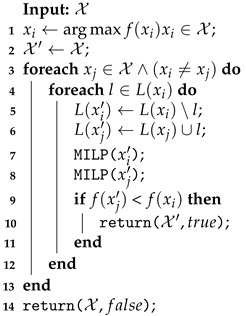


The Shift function not only performs the inter-route changes but also returns the first improved solution obtained. Since this function tries to minimize the longest route, the comparison between $f(x_{j}^{\prime })$ and $f(x_{i})$ presented in (line 9) evaluates the flight time cost. The same evaluation is performed in the Swap and UAV Swap function. The Recharging Station Shutdown evaluates the number of recharging stations in the solution.

As this work deals with the multiobjective problem, the neighborhood functions designed to minimize the longest route cost may also interfere with the other objective modifying the number of recharging stations. In this case, these neighborhood functions only change the incumbent solution if the route cost is improved and inserted into the structure of all the nondominated solutions. The update function on MOVNS evaluates the nondominated solutions found by the neighborhood functions on VND (see line 8 in Algorithm 1).

The Swap function is structurally similar to the Shift. However, it acts to exchange the coverage rows between $x_{i}^{\prime }$ and $x_{j}^{\prime }$. For this reason, the route evaluation (line 9) in Algorithm 6 must be modified in Swap to verify if one of the routes improves the solution $\max\{f\left(x_{i}^{\prime }\right),f\left(x_{j}^{\prime }\right)\}$ < $f(x_{i})$.

The UAV Swap procedure exchanges the vehicles between two routes with different models. As summarized in Algorithm 7, the routes with different UAVs $m\left(x_{i}\right)\neq m\left(x_{j}\right)$ (line 3) have their vehicles exchanged (lines 4–5). The MILP solver is then applied to reroute the clusters with the new vehicles (lines 6–7). This new solution $X^{\prime }$ and the value true are returned if the longest route’s cost is lower than the cost of the current longest one (lines 8–10). Unlike the other neighborhood functions presented here (Shift and Swap), changing the UAV models is not restricted to improving the maximum cost route because it is expected that this route has already been assigned to the best-performing vehicle.

To shut down a recharging station on EMR, the function described in Algorithm 8 is applied. This function has as the input the current solution X, and the set of all recharging stations opened $S_{all}$ in the solution (line) is defined in its initialization. At any given iteration (lines 4–14), the feasibility of the shutting down operation of a recharging station is checked. For this, each route $x_{i}\in X$ with the station $s\in S(x_{i})$ is rerouted without the station (lines 6–9). The MILP function (line 8) assigns infinity to the unfeasible cases where the vehicle does not have sufficient battery to cover its rows. If all routes in $X^{\prime }$ are feasible, the set of all recharging stations $S_{all}^{\prime }$ is updated (line 11), and the new solution $X^{\prime }$ and the true status are returned (line 12). If no recharging station was shuttered, the input solution X is returned with the value false to the improved variable in the VND (line 15).
**Algorithm 7:** Unmanned air vehicles (UAV) Swap performed on EMR.
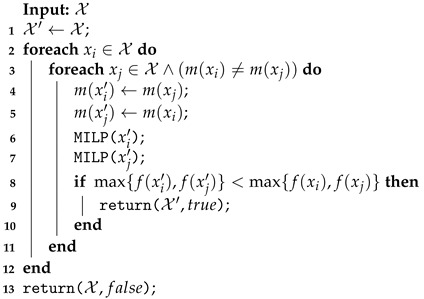
**Algorithm 8:** Recharging Station Shutdown performed on EMR.
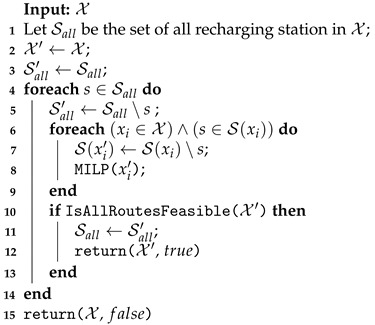


#### 5.3.2. Other Approaches

As EMR calls the exact method to route each change performed by its neighborhood functions on the clusters, we propose three other approaches that aim to reduce these calls. Unlike the EMR, which first builds the clusters using heuristics and then applies the exact method to the route (cluster first-route, second approach), these new approaches are classified as improvement heuristics, as stated in [73]. In this case, a heuristic was implemented to route the new clusters, and the exact method was executed only at different steps of the algorithm, not on every new route generated by the VND.

The first approach proposed is the exact method applied selectively (EMAS), which uses the exact method to improve only the solution returned by the routing heuristic. In this case, its neighborhood functions build new routes, evaluate them, check their feasibility, and then select one to be improved by the exact method.

The Shift and Swap functions use the routing heuristic to compute the routes and select the best route among the longest routes to be improved by the exact method. The Swap function is described in Algorithm 9. After exchanging rows between routes (lines 8–11), a heuristic is applied to recalculate such routes (lines 12–13). If the row swap produces a better solution than the incumbent one, then the exact method is applied to improve only these two routes, and the solution $X^{\prime }$ with the value true, indicating that an improvement was found, is returned (lines 14–17). Otherwise, the best heuristic solution is assigned to the $X^{\prime \prime }$, lines 19–24. Not finding a better solution than the current one, the MILP is applied to the best routing heuristic results (lines 28–29). This solution $X^{\prime \prime }$ and the value true are returned if it is better than the incumbent (lines 30–32). The EMAS Shift function algorithm is similar to this Swap function, different only in the row shift displacement (removing lines 10–11 from Algorithm 9).
**Algorithm 9:** Swap performed in EMAS.
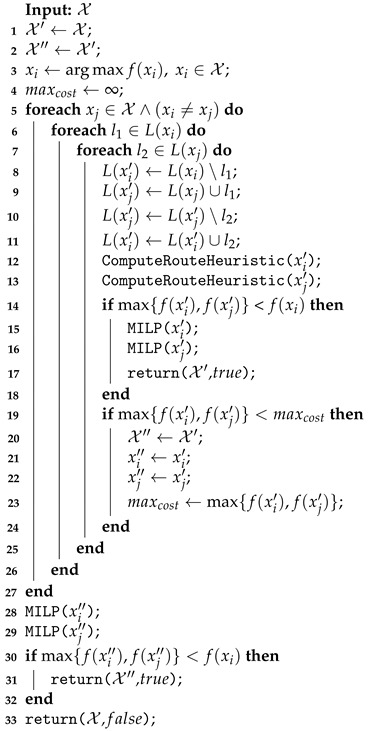


The ComputeRouteHeuristic (lines 12–13) is applied to compute the changed route in the EMAS neighborhood functions (Shift and Swap) and is used in the other two approaches, EMNF and WEM. This heuristic builds the new route based on the current one, rearranging each coverage row using removal and insertion procedures.

**Figure 7:**
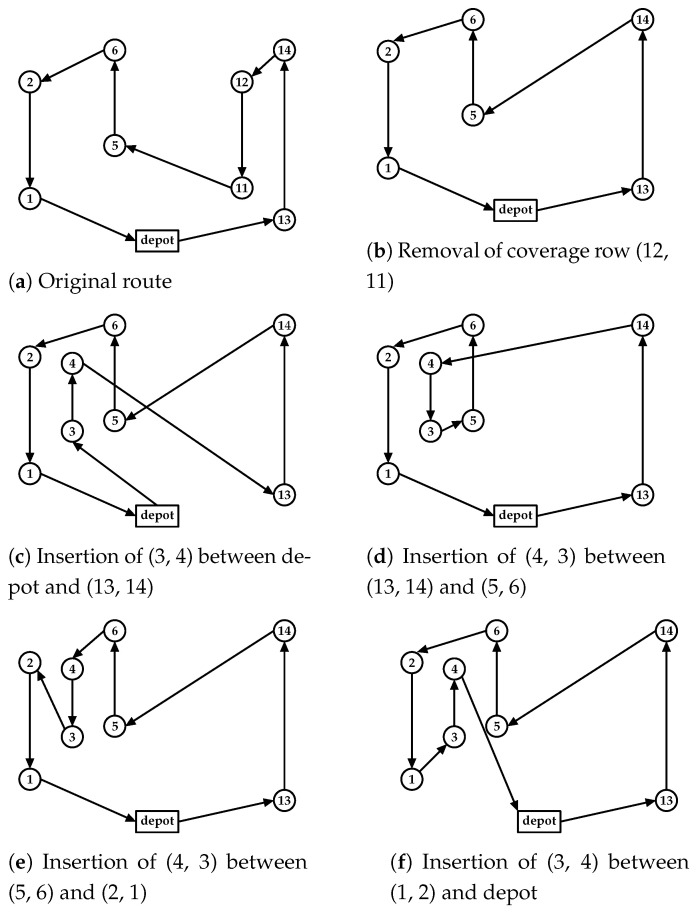
Example of link evaluation heuristic performed on Swap to compute the route.

The removal operation deletes the vertices and all links associated with a selected coverage row, adding a new path linking its predecessor and successor vertices. This new path must be a feasible route, ensuring that the UAV has enough charge to reach the next recharging station or depot from the successor vertex (e.g., in Figure 7b, the removal of the coverage row (11, 12) deletes its vertices and the edges (14, 12), (12, 11), and (11, 5) from the route). A path search algorithm is used to link the vertices (14–5) considering the vehicle constraint, ensuring that the UAV can reach the next recharging station or depot after the successor vertex (vertex 5).

The insertion operation adds the coverage row vertices into the route and evaluates different connections by interchanging the order in which the depot or the other coverage rows are linked to this new row. The insertion procedure generates a new route for each feasible linkage in its initial step, as shown in Figure 7c–f. Here, the original coverage row directions are preserved, only adjusting the inserted row direction to fit the route, as shown in Figure 7b. To build the route in Figure 7c, the edge to connect the first coverage row in Figure 7b (depot, 13) is removed and the row (3, 4) is inserted. The new coverage row direction is defined by the nearest vertex of the depot (vertex 3), and a feasible path connecting the vertex (4, 13) is evaluated and inserted to close the route (in this case, a direct edge). This process continues changing the connection order, as shown in Figure 7d–f (see Figure 7d, where the inserted row (4, 3) is the second row visited in the route, Figure 7e is the third, and so on). After evaluating all row interchanges, the lowest-cost route is selected.

The row interchange considers the remaining UAV flight time to connect the inserted row in the route. If the direct link violates this constraint, the path search algorithm is applied to find the input and output links of this new coverage row (as previously stated, its direction is set by the nearest vertex to connect to the route). A complete subgraph is built by connecting its initial and final vertices to all available recharging stations in both cases. A search algorithm is then applied to find a path that connects both vertices considering the UAV flight time capacity, and recharges closer to the final vertex, aiming to reach the next route segment with the maximum amount of charge. Once found, its feasibility is evaluated, checking if the UAV can reach the next recharging station or the depot after the inserted path.

**Figure 8:**
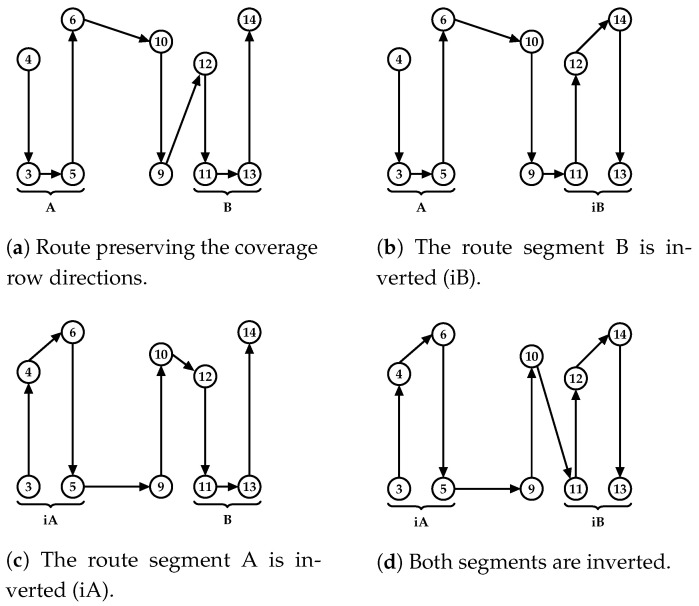
Example of routes generated by changing the coverage row directions.

The first step of the insertion procedure, intended to preserve the route’s original structure by not changing its coverage row directions, can be a suitable strategy to generate routes with approximate costs of the original route, especially when exchanging rows similar in lengths and vertex alignments. However, as the routes can have quite different rows, a second step to the insertion procedure is proposed to evaluate the route cost by changing its row directions, as shown in Figure 8. First, in Figure 8a, as explained before, the insertion of the new row (10,9) preserves the other row directions. Here, these rows are organized into two route segments (A and B). Then, in this second step of the insertion procedure, the same linkage process is performed, combining the inversion of these segments, as illustrated in Figure 8b–d. If the new row is inserted at the beginning or end of the route, only one segment is built (i.e., segment A), resulting in two routes evaluated with (A and iA) row directions.

For all routes generated by the insertion procedure, only the best one is returned. If no route is generated (all routes are infeasible), the heuristic continues evaluating the next coverage row change performed by the current neighborhood function (i.e., Shift or Swap).

The UAV Swap function applied to EMAS is described in Algorithm 10. In this case, after the UAV model change (lines 4–5), the route edge costs (flight time) are updated considering the vehicle performance, and the route feasibility is verified by checking the capacity of the vehicle to cover its new route (lines 6–7). The improved solution, if found, is returned together with the value true, flagging it as a better solution (lines 8–10).
**Algorithm 10:** UAV Swap performed on EMAS, EMNF, and WEM approaches.
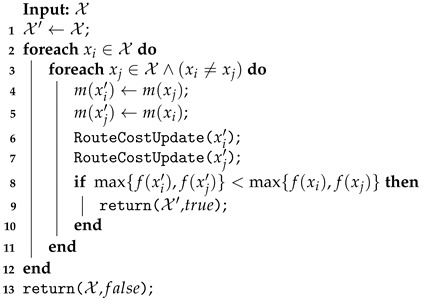


The Recharging Station Shutdown on the EMAS neighborhood function is shown in Algorithm 11. As explained for EMR, each iteration must check the feasibility of removing a station from all routes containing them. However, in EMAS, instead of applying the exact method to reroute, a procedure checks whether it is possible to deviate the recharging to the nearest station in relation to the one under consideration for shutdown (line 8). The station is shut down if all vehicles directed to it can perform this deviation (lines 10–16). In this case, the MILP model is applied to improve the feasible solution (line 12).
**Algorithm 11:** Recharging Station Shutdown performed on EMAS.
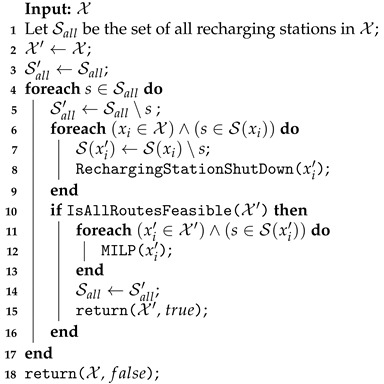


All these modifications designed to the EMAS neighborhood functions are also used on EMNF and WEM. The difference is that the EMAS uses the exact method on most neighborhood functions to improve the selected routes. In the EMNF, the exact method is removed from the neighborhood functions and added to the VND as a neighborhood function, acting as an intraroute operator performed on all incumbent solution routes. Conversely, the WEM does not apply the exact method in any part of its neighborhood structure. It is a matheuristic classified as a one-shot approach that applies the exact method only in the initialization [73].

## 6. Experimental Results

This section presents the experimental results from the evaluation of the matheuristics proposed in this paper. The algorithms and MILP formulations were implemented using C++ and compiled with GCC 4.8.5. The MILP was solved with Gurobi version 8.1.1 [74], using the default values. All experiments were performed on an Intel(R) Core(TM) i7 2.8 GHz computer with 8 GB RAM.

### 6.1. Test Problems and Performance Metrics

**Figure 9:**
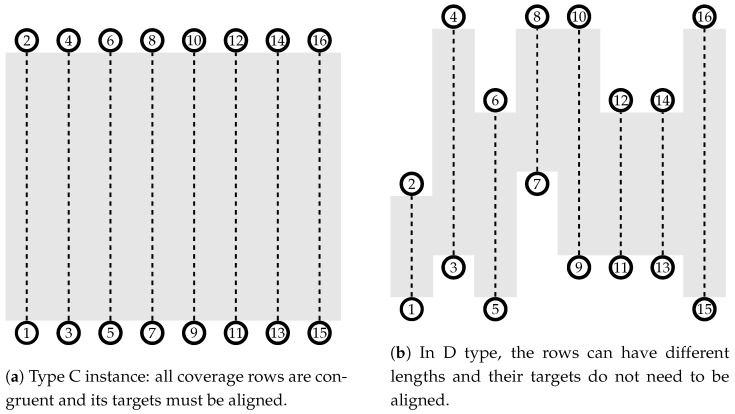
Examples of type C and D instances.

The performance of the MOVNS variations was evaluated using an experimental set-up considering 22 problem instances ranging from 20 to 60 targets plus the same number of recharging stations and one depot, being the number of vertices of the instance |V|=2×|T|+1. The size of the fleet used to cover the area varies with {3,6} UAVs for instances including up to 50 targets. For 60 targets, the possibility of using nine vehicles resulted in fleets with {3,6,9} UAVs. Two classes of problems, as shown in Figure 9, were analyzed: type C (Congruent) has vertically aligned targets in the plane, whereas type D (Different) problems have targets oscillating on the y-axis. The travel cost is given by the time spent to move from vertex *i* to vertex *j*, considering the distance between these vertices and the flight speed of the UAV ($c_{i,j}^{k}=\gamma_{i,j}/v_{k}$). Regardless of the problem class (C or D), the flight range to perform a row coverage was randomly defined between upper and lower bound values considering the lowest-capacity UAV model, forcing the vehicles to recharge to complete the task. The range upper bound was defined to ensure that the UAV could cover any rows without a recharge, considering a fully charged battery. The lower bound was set to half of the upper bound value. The coverage area of the instances varied from 66.5 km${}^{2}$ (20 targets) to 203.1 km${}^{2}$ (60 targets) in C types instances and 58.5 km${}^{2}$ (20 targets) to 151 km${}^{2}$ (60 targets) in type D.

All coverage missions were performed by heterogeneous fleets with three UAV models. Model 1 is able to fly for $F_{1}=$ 1800 s with flight speed $v_{1}=$ 16 m/s and recharging time with flight-time ratio of $q_{1}=2$. Model 2 vehicles have a fuel capacity to fly for $F_{2}=$ 1320 s with flight speed of $v_{2}=$ 15 m/s and $q_{2}=2.73$. The UAVs of model 3 have the lowest coverage performance, flying for $F_{3}=$ 1200 s at $v_{3}=$ 15 m/s and with $q_{3}=3$. The fleets were all balanced: each one had the same number of vehicles of each model (e.g., a fleet with six UAVs had two vehicles of the same model).

These instances were solved using the following proposed approaches: EMR, EMAS, EMNF, and WEM. After preliminary tests, the constant $i_{max}$ of these algorithms was set to $i_{max}=50$ (see Algorithm 1, line 5).

The relative performance of these approaches was assessed using the following metrics:

- Execution time: the computation time required for the algorithm to return the front.
- Cardinality: the number of solutions contained in the front.
- Hypervolume: estimates the proximity of the solutions to the Pareto optimal front. The hypervolume is given by the sum of the hypercubes formed by the nondominated solutions obtained by the algorithms. It calculates the coverage region volume between the solution points at the front and a given reference point. Although the Pareto optimal front is not known for the problem addressed in this paper, the hypervolume helps to measure the quality of one solution in relation to another, where larger hypervolumes indicate better solutions [75]. In this work, the hypervolume (S) was independently normalized to the interval (0, 1) for each problem.
- Coverage: measures the quality of one front in relation to another. Given two approximate Pareto fronts A and B, the coverage metric (C(A, B)) calculates the number of B solutions that are weakly dominated by another A front given B’s cardinality. If C(A, B) is equal to 1, all B solutions are dominated by A front solutions. If the value returned is equal to 0, it indicates the opposite situation in which A dominates none of B’s solutions [75]. A generalized version of the coverage of two sets, called coverage of many sets, is also used to simplify reporting the relative coverage values [76]. Instead of quantifying the amount by which one given algorithm covers another, this generalized metric measures how much a given algorithm covers the union of the final fronts returned by all algorithms, except itself.

Both the hypervolume and coverage metrics return values representing percentages, whereas the cardinality metric provides absolute values.

### 6.2. Statistical Design

We employed statistical tests designed to detect significant differences among the proposed approaches and to estimate their magnitude considering each quality metric. The data used for this comparison included the values for the given metrics calculated for the final Pareto fronts obtained on 15 independent trial runs of each algorithm on each problem.

For each metric, the experiment was designed as a randomized complete block design (RCBD) with the algorithms as the levels of the experimental factor and the problems as blocking factors [77]. By treating the problems as blocks, it was possible to model and remove the effects of different instances in the algorithm’s performance and obtain an overall performance difference across all considered test instances. The null hypothesis of an absence of differences among the algorithms evaluated on all problems was considered against two-sided alternatives. For this experimental analysis, a significance level of 95% (α=0.05) was considered.

To avoid assumptions of the F-test, the Friedman test was used [78,79]. In this case, the rejection of the null hypothesis implies that there is a significant difference among the implemented algorithms in the metric evaluated.

After the Friedman test indicates significance, the Nemenyi’s post-hoc test was applied to identify differences between the algorithms [80]. Then, for the pairs of algorithms indicated as significantly different, the magnitude of the effect size was calculated by the Hodges–Lehman estimator [81]. To perform an independent estimation of the effect size for each algorithm, the estimators of the effect of the instance (block) were calculated using the least squares and removed from the samples.

### 6.3. Results and Discussion

The results obtained for the experimental comparison are summarized in Table 2, which reports the mean and standard deviation values of the metrics (execution time (s), coverage of many sets (CS), cardinality (Card), and hypervolume (S)) considered for each problem; and Table 3 summarizes the results of the statistical analysis presenting the magnitude of the statistically significant differences.

As observed in Table 2, the WEM approach outperformed the others in the execution time metric, and, as expected, the EMR was the slowest. The analysis in Table 3 supports this observation, with WEM providing a reduction of $8.96\times 10^{4}$ s compared to EMR, $1.88\times 10^{2}$ s in relation to EMAS, and $1.2\times 10^{3}$ s in comparison to EMNF. The results indicated no statistically significant difference between EMAS and EMNF for this metric.

The best results for the coverage metric observed in Table 2 were mostly obtained by EMR, with a few instances where EMNF outperformed it. However, by examining the results in Table 3, we observed no statistically significant differences in performance between these two algorithms. EMR outperformed WEM by 0.73 and EMAS by 0.41. These results are robust indicators of the superiority of the fronts returned by EMR, which dominated 73% of the ones computed by WEM and 41% of those obtained by EMAS. The quality of the front from EMNF outperformed only WEM, dominating about 46% of the fronts returned by the last one.

The performances of EMR, EMAS, and EMNF were quite similar in terms of the cardinality metric. Table 3 shows that, on average, these algorithms were only slightly different. Table 3 indicates that the EMR returned 1.6 more solutions than EMAS and 2.2 more than WEM. The variation in performance between EMAS and EMNF was somewhat modest, confirmed by the lack of statistically significant differences. EMNF outperformed WEM, returning 1.7 more solutions.

For the hypervolume, all algorithms presented consistent performance in most problems, as shown in Table 2, with WEM being a little worse in terms of solution quality compared to the other three methods. The statistical analysis reported in Table 3 confirms this observation, showing that only WEM had statistically significant differences among EMR, EMAS, and EMNF. EMR was 17% superior, EMAS was 10%, and EMNF enhanced the quality of WEM solutions by around 12%. Due to the statistical results, we cannot conclude that a difference exists among EMR, EMAS, and EMNF. This means that EMAS and EMNF were, in general, able to return points in their fronts closer to those in the EMR front.

**Figure 10:**
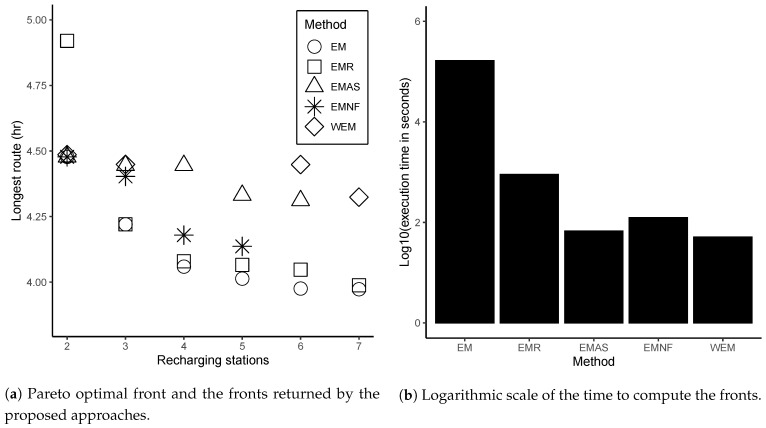
Results for a type D instance with 20 targets and 3 UAVs.

As the cost of generating the exact Pareto-optimal fronts to all instances used in this work was prohibitive, we chose one of the smallest instances, type D with 20 targets and three UAVs, to illustrate the performance of the exact approach and the proposed methods (Figure 10). The fronts obtained by these methods, including the exact one referenced as EM, are shown in Figure 10a. The fronts of EMR, EMAS, EMNF, and WEM were randomly selected among their replications.

A common method used to generate the Pareto-optimal front is transforming the multiobjective problem into several mono-objective problems, where the optimal solutions of these problems represent the Pareto front solutions. In this work, we used two strategies to generate the optimal points belonging to the set of efficient solutions: the weighted problem $P_{\lambda }$ and the problem ϵ-restrict $P_{\epsilon }$ [82]. The $P_{\lambda }$ approach aggregates the different objectives through the weighted sum. $P_{\epsilon }$ is based on scalarization, which minimizes one of the objectives while restrictions limit the other objectives.

First, the $P_{\lambda }$ was applied to aggregate both objectives of our problem by the weighted sum. The parameter was set to λ={0,1} to obtain the extreme points of the front. In this case, λ=0 (minimize only the number of stations) returns a solution with two recharging stations and the longest route with 4.5 h, and for λ=1 (minimize only the longest route), another extreme point, returns seven recharging stations and longest route with 3.98 h.

The other points in the Pareto front were calculated by the ϵ-restrict approach, minimizing the longest route while the number of recharging stations was constrained by an integer constraint ranging from 3 to 6. Figure 10b shows the computation time in the logarithmic scale for each method to generate the solutions in the front. For this instance, the exact method (EM) calculates the Pareto-optimal front as 45.68 h and WEM was the fastest method, returning its front in 51 s.

Given the high computational costs of the exact method, we implemented the EMR (cluster first-route second) matheuristic that combines the MOVNS with an MILP formulation to generate the approximated front. This method splits the original problem into small problems, one for each UAV, and applies the exact method for routing into the neighborhood functions. The results shown in Table 2 indicate that EMR was the best in terms of the quality of the solutions in most cases. However, it was the most time-consuming method.

The decrease in the number of calls of the exact method in EMAS, EMNF, and WEM was achieved by designing a heuristic to route and evaluating each change performed by their neighborhood functions. The strategy adopted in EMNF and EMAS to mitigate the trade-off between execution time and the quality of the computed fronts was a wise application of the exact method to improve some solutions. The EMAS calls the exact method into the neighborhood functions only to the best solution returned by the routing heuristic. Conversely, EMNF adds an intraroute operator on its VND that uses the exact method to improve each route of the current solution separately.

The statistical tests showed that EMNF was faster than EMR and superior to WEM considering cardinality, coverage, and hypervolume metrics. The WEM approach was the fastest as the exact method is not used on its VND. The statistical analysis indicated a significant degradation in all metrics used to evaluate the returned fronts compared to EMR and EMNF. However, between WEM and EMAS, no significant differences were detected in coverage and cardinality.

Although the results in Table 2 show EMAS was faster than EMNF in almost instance problems, and most EMNF results were superior to EMAS for the other metrics, no statistically significant differences were observed between them for any metric.

## 7. Conclusions

This paper proposed strategies for the multiobjective location-routing problem in complete area coverage missions using heterogeneous UAV fleets. Here, the decision on the recharging station locations and the number of stations to be opened are jointly considered with UAVs routing criteria. As the cost to compute the Pareto-optimal front to this problem is usually prohibitive, four matheuristics based on MOVNS were presented, evaluated, and compared using four performance metrics (execution time, coverage, cardinality, and hypervolume).

The proposed EMR, a cluster first-route second matheuristic, returned most of the best results considering the fronts quality metrics. However, it performed the worst in execution time. The other approaches, EMAS, EMNF, and WEM, significantly reduced the intensity of calls of the exact method using heuristics in the routing of clusters. Our experimental comparison showed that the EMNF presented relatively high-quality performance regarding the front’s quality metrics compared to EMAS and WEM. The statistical analysis results indicated no significant degradation compared to EMR, standing out for applying the exact method only as a neighborhood function in the VND, where it acts like an intraroute operator.

Conversely, as expected, WEM, a one-shot approach where the MILP is only solved in the beginning, produced superior performance in terms of the execution time. Despite the results showing a significant degradation in the quality of its fronts compared to EMR and EMNF, no evidence of significant difference from EMAS in coverage or cardinality was observed. A modest loss in hypervolume was verified. Thus, WEM can be indicated for applications that tolerate the observed degradation in the quality of its solution in order to speed up computation.

In the future, we intend to consider the recharging station’s capability to attend a limited number of UAVs. In this case, the recharging stations could be modeled as a queuing system, as proposed in [40]. We also plan to adopt some strategies to avoid collisions in the presence of intersecting routes. For this, it would be necessary to implement some speed planner, as presented in [20,22].

## Figures and Tables

**Table 1:** Differences among the proposed approaches.

Exact Method Application in the VND	Approaches			
	EMR ${}^{1}$	EMAS ${}^{2}$	EMNF ${}^{3}$	WEM ${}^{4}$
every routing call	X			
only in the best heuristic solution		X		
intraroute like a neighborhood function			X	
not applied				X

^1^ Exact Method for Routing. ^2^ Exact Method Applied Selectively. ^3^ Exact Method as Neighborhood Function. ^4^ Without Exact Method.

**Table 2:** Mean values and standard deviations (in the parentheses) for the execution time, coverage, cardinality, and hypervolume metrics.

Type	Target	UAV	Execution Time (sec.)				Coverage (CS)				Cardinality (Card)				Hypervolume (S)			
			EMR	EMAS	EMNF	WEM	EMR	EMAS	EMNF	WEM	EMR	EMAS	EMNF	WEM	EMR	EMAS	EMNF	WEM
C	20	3	$5.07\times 10^{2}$ ($1.18\times 10^{2}$)	$4.16\times 10^{1}$ ($1.81\times 10^{1}$)	$4.94\times 10^{1}$ ($2.24\times 10^{1}$)	$1.56\times 10^{1}$ ($0.72\times 10^{1}$)	0.86 (0.13)	0.59 (0.15)	0.71 (0.22)	0.20 (0.26)	2.60 (0.49)	1.93 (0.44)	2.07 (0.25)	1.53 (0.50)	1.0 (0.00)	0.98 (0.04)	0.98 (0.04)	0.74 (0.25)
		6	$4.59\times 10^{1}$ ($2.98\times 10^{1}$)	$0.70\times 10^{1}$ $\left(0.35\times 10^{1}\right)$	$1.37\times 10^{1}$ ($1.68\times 10^{1}$)	$0.21\times 10^{1}$ $\left(8.02\times 10^{1}\right)$	0.92 (0.19)	0.35 (0.23)	0.30 (0.34)	0.50 (0.23)	2.27 (0.44)	2.47 (0.88)	1.73 (0.77)	2.20 (0.75)	0.84 (0.08)	0.64 (0.15)	0.54 (0.24)	0.66 (0.15)
	30	3	$1.11\times 10^{4}$ ($2.04\times 10^{3}$)	$3.90\times 10^{2}$ $\left(1.73\times 10^{2}\right)$	$7.58\times 10^{2}$ $\left(1.99\times 10^{2}\right)$	$1.10\times 10^{2}$ $\left(3.13\times 10^{1}\right)$	0.5 (0.24)	0.76 (0.18)	0.84 (0.22)	0.10 (0.11)	4.40 (0.49)	4.07 (0.68)	4.07 (0.25)	3.40 (0.71)	0.96 (0.02)	0.98 (0.02)	0.99 (0.02)	0.77 (0.06)
		6	$4.31\times 10^{3}$ ($1.55\times 10^{3}$)	$1.62\times 10^{2}$ ($5.75\times 10^{1}$)	$2.15\times 10^{2}$ ($1.51\times 10^{2}$)	$5.03\times 10^{1}$ $\left(2.03\times 10^{1}\right)$	0.92 (0.11)	0.47 (0.17)	0.28 (0.14)	0.13 (0.10)	(4.60) (0.71)	3.80 (1.11)	3.93 (1.39)	3.00 (0.73)	(0.99) (0.01)	0.94 (0.03)	0.91 (0.05)	0.88 (0.04)
	40	3	$3.69\times 10^{4}$ $\left(9.95\times 10^{3}\right)$	$1.66\times 10^{3}$ $\left(3.67\times 10^{2}\right)$	$2.71\times 10^{3}$ $\left(3.41\times 10^{2}\right)$	$6.87\times 10^{2}$ $\left(2.36\times 10^{2}\right)$	0.54 (0.12)	0.42 (0.10)	(0.85) (0.10)	0.05 (0.05)	6.87 (0.81)	6.67 (1.40)	7.60 (0.71)	6.33 (1.96)	0.97 (0.01)	0.96 (0.02)	0.99 (0.00)	0.91 (0.03)
		6	$2.17\times 10^{4}$ $\left(7.45\times 10^{3}\right)$	$2.47\times 10^{2}$ $\left(9.89\times 10^{1}\right)$	$4.73\times 10^{2}$ $\left(2.11\times 10^{2}\right)$	$8.74\times 10^{1}$ $\left(3.82\times 10^{1}\right)$	0.90 (0.14)	0.43 (0.11)	0.48 (0.22)	0.12 (0.12)	4.60 (0.71)	3.27 (0.57)	3.40 (0.61)	3.27 (1.00)	0.98 (0.02)	0.80 (0.03)	0.84 (0.07)	0.67 (0.07)
	50	3	$1.80\times 10^{5}$ $\left(5.40\times 10^{4}\right)$	$3.83\times 10^{3}$ $\left(1.32\times 10^{3}\right)$	$1.23\times 10^{4}$ $\left(2.97\times 10^{3}\right)$	$1.29\times 10^{3}$ $\left(4.76\times 10^{2}\right)$	0.49 (0.12)	0.43 (0.09)	0.76 (0.14)	0.04 (0.04)	9.53 (0.81)	7.27 (1.06)	9.33 (1.35)	5.93 (1.39)	0.91 (0.02)	0.94 (0.02)	0.96 (0.01)	0.88 (0.02)
		6	$1.22\times 10^{5}$ $\left(3.98\times 10^{4}\right)$	$4.90\times 10^{2}$ $\left(2.08\times 10^{2}\right)$	$2.59\times 10^{3}$ $\left(7.60\times 10^{2}\right)$	$1.38\times 10^{2}$ $\left(6.62\times 10^{1}\right)$	0.93 (0.07)	0.19 (0.11)	0.36 (0.11)	0.02 (0.04)	8.40 (1.58)	3.33 (1.14)	5.47 (1.26)	2.53 (1.15)	0.96 (0.02)	0.90 (0.03)	0.94 (0.01)	0.86 (0.03)
	60	3	$2.46\times 10^{5}$ $\left(6.16\times 10^{4}\right)$	$8.52\times 10^{3}$ $\left(2.57\times 10^{3}\right)$	$2.04\times 10^{4}$ $\left(6.30\times 10^{3}\right)$	$3.41\times 10^{3}$ $\left(1.94\times 10^{3}\right)$	0.52 (0.17)	0.43 (0.12)	**0.76** (**0.17**)	0.02 (0.02)	7.47 (0.96)	7.60 (1.02)	**8.13** (**0.96**)	5.07 (1.53)	0.93 (0.02)	0.96 (0.00)	**0.97** (**0.00**)	0.87 (0.03)
		6	$3.21\times 10^{5}$ $\left(1.60\times 10^{5}\right)$	$1.14\times 10^{3}$ $\left(4.02\times 10^{2}\right)$	$5.90\times 10^{3}$ $\left(4.00\times 10^{3}\right)$	$2.37\times 10^{2}$ $\left(9.80\times 10^{1}\right)$	0.59 (0.22)	0.32 (0.18)	**0.64** (**0.25**)	0.04 (0.06)	**8.20** (**1.60**)	4.33 (1.30)	5.93 (1.91)	3.27 (1.12)	**0.91** (**0.06**)	0.84 (0.03)	0.89 (0.05)	0.71 (0.05)
		9	$7.04\times 10^{4}$ $\left(2.19\times 10^{4}\right)$	$2.94\times 10^{2}$ $\left(1.09\times 10^{2}\right)$	$1.40\times 10^{3}$ $\left(1.30\times 10^{3}\right)$	$7.73\times 10^{1}$ $\left(6.07\times 10^{1}\right)$	**0.91** (**0.10**)	0.30 (0.18)	0.43 (0.16)	0.05 (0.08)	**5.13** (**1.02**)	3.40 (0.88)	3.73 (1.29)	2.60 (1.54)	**0.95** (**0.05**)	0.69 (0.06)	0.78 (0.07)	0.48 (0.08)
	Mean		$9.22\times 10^{4}$ $\left(3.24\times 10^{4}\right)$	$1.52\times 10^{3}$ $\left(4.84\times 10^{2}\right)$	$4.25\times 10^{3}$ $\left(1.48\times 10^{3}\right)$	$5.55\times 10^{2}$ $\left(2.70\times 10^{2}\right)$	**0.73** (**0.15**)	0.43 (0.15)	0.58 (0.19)	0.12 (0.10)	**5.82** (**0.87**)	4.38 (0.95)	5.04 (0.98)	3.56 (1.12)	**0.95** (**0.03**)	0.87 (0.04)	0.89 (0.05)	0.77 (0.07)
D	20	3	$4.70\times 10^{2}$ $\left(3.96\times 10^{2}\right)$	$9.21\times 10^{1}$ $\left(4.65\times 10^{1}\right)$	$1.01\times 10^{2}$ $\left(4.40\times 10^{1}\right)$	$4.21\times 10^{1}$ $\left(1.79\times 10^{1}\right)$	**0.66** (**0.20**)	0.63 (0.25)	0.56 (0.22)	0.13 (0.11)	3.13 (1.54)	3.33 (0.70)	3.13 (0.50)	**3.60** (**0.95**)	0.88 (0.06)	**0.90** (**0.06**)	0.89 (0.06)	0.79 (0.07)
		6	$4.90\times 10^{1}$ $\left(2.22\times 10^{1}\right)$	$2.38\times 10^{1}$ $\left(0.80\times 10^{1}\right)$	$2.09\times 10^{1}$ $\left(1.66\times 10^{1}\right)$	$0.95\times 10^{1}$ $\left(0.33\times 10^{1}\right)$	**0.86** (**0.16**)	0.40 (0.25)	0.32 (0.27)	0.45 (0.19)	2.00 (0.00)	2.53 (0.72)	2.27 (0.57)	**3.13** (**0.88**)	**0.86** (**0.01**)	0.78 (0.08)	0.78 (0.06)	0.83 (0.04)
	30	3	$1.20\times 10^{4}$ $\left(4.71\times 10^{3}\right)$	$9.76\times 10^{2}$ $\left(2.40\times 10^{2}\right)$	$1.35\times 10^{3}$ $\left(5.11\times 10^{2}\right)$	$2.55\times 10^{2}$ $\left(8.12\times 10^{1}\right)$	**0.72** (**0.12**)	0.51 (0.15)	0.62 (0.11)	0.01 (0.02)	5.00 (0.94)	6.40 (0.80)	**7.20** (**1.33**)	5.67 (1.25)	**0.87** (**0.04**)	0.85 (0.04)	0.86 (0.03)	0.60 (0.05)
		6	$1.88\times 10^{4}$ $\left(3.89\times 10^{3}\right)$	$1.47\times 10^{2}$ $\left(6.24\times 10^{1}\right)$	$3.01\times 10^{2}$ $\left(1.27\times 10^{2}\right)$	$6.26\times 10^{1}$ $\left(3.87\times 10^{1}\right)$	**0.98** (**0.05**)	0.25 (0.08)	0.35 (0.08)	0.03 (0.06)	**10.47** (**0.72**)	4.00 (1.10)	3.73 (0.85)	4.47 (1.45)	**0.99** (**0.01**)	0.85 (0.03)	0.86 (0.03)	0.77 (0.04)
	40	3	$8.25\times 10^{4}$ $\left(3.75\times 10^{4}\right)$	$4.04\times 10^{3}$ $\left(1.72\times 10^{3}\right)$	$9.58\times 10^{3}$ $\left(2.74\times 10^{3}\right)$	$1.22\times 10^{3}$ $\left(4.82\times 10^{2}\right)$	**0.65** (**0.00**)	0.39 (0.02)	0.55 (0.10)	0.01 (0.00)	9.69 (1.69)	7.73 (1.73)	**11.13** (**3.14**)	6.53 (2.42)	0.87 (0.04)	0.91 (0.02)	**0.93** (**0.02**)	0.84 (0.03)
		6	$2.74\times 10^{4}$ $\left(5.63\times 10^{3}\right)$	$6.08\times 10^{2}$ $\left(1.76\times 10^{2}\right)$	$1.55\times 10^{3}$ $\left(6.70\times 10^{2}\right)$	$1.69\times 10^{2}$ $\left(7.23\times 10^{1}\right)$	**0.99** (**0.03**)	0.32 (0.18)	0.42 (0.13)	0.08 (0.09)	**6.33** (**0.47**)	4.93 (1.34)	5.73 (1.69)	4.87 (1.20)	**0.99** (**0.01**)	0.87 (0.04)	0.90 (0.04)	0.79 (0.03)
	50	3	$9.65\times 10^{5}$ $\left(2.60\times 10^{4}\right)$	$7.47\times 10^{3}$ $\left(1.67\times 10^{3}\right)$	$3.06\times 10^{4}$ $\left(4.67\times 10^{3}\right)$	$2.78\times 10^{3}$ $\left(8.44\times 10^{2}\right)$	**0.77** (**0.08**)	0.37 (0.07)	0.56 (0.08)	0.00 (0.01)	12.00 (1.21)	9.47 (1.31)	**13.40** (**1.45**)	9.27 (2.05)	0.88 (0.02)	0.92 (0.01)	**0.94** (**0.02**)	0.84 (0.03)
		6	$2.38\times 10^{5}$ $\left(8.13\times 10^{4}\right)$	$1.31\times 10^{3}$ $\left(4.59\times 10^{2}\right)$	$5.51\times 10^{3}$ $\left(1.67\times 10^{3}\right)$	$5.17\times 10^{2}$ $\left(1.42\times 10^{2}\right)$	**0.90** (**0.06**)	0.32 (0.08)	0.49 (0.08)	0.00 (0.00)	**10.73** (**1.91**)	7.13 (1.59)	8.87 (2.09)	7.20 (1.87)	**0.93** (**0.02**)	0.86 (0.02)	0.89 (0.03)	0.75 (0.03)
	60	3	$1.64\times 10^{6}$ $\left(3.78\times 10^{5}\right)$	$2.23\times 10^{4}$ $\left(5.94\times 10^{3}\right)$	$8.41\times 10^{4}$ $\left(9.96\times 10^{3}\right)$	$7.92\times 10^{3}$ $\left(2.61\times 10^{3}\right)$	**0.86** (**0.05**)	0.26 (0.10)	0.56 (0.06)	0.00 (0.01)	**15.87** (**1.09**)	9.60 (2.18)	15.80 (1.83)	9.93 (2.93)	0.96 (0.00)	0.87 (0.02)	**0.97** (**0.01**)	0.68 (0.07)
		6	$2.45\times 10^{5}$ $\left(1.52\times 10^{5}\right)$	$1.80\times 10^{3}$ $\left(4.09\times 10^{2}\right)$	$4.18\times 10^{3}$ $\left(2.49\times 10^{3}\right)$	$7.65\times 10^{2}$ $\left(2.25\times 10^{2}\right)$	**0.82** (**0.18**)	0.29 (0.11)	0.48 (0.20)	0.00 (0.00)	**6.53** (**2.00**)	4.67 (1.07)	5.73 (2.52)	4.07 (1.12)	**0.92** (**0.03**)	0.85 (0.02)	0.88 (0.06)	0.68 (0.05)
		9	$3.24\times 10^{5}$ $\left(7.41\times 10^{4}\right)$	$8.22\times 10^{2}$ $\left(2.91\times 10^{2}\right)$	$4.33\times 10^{3}$ $\left(2.07\times 10^{3}\right)$	$3.23\times 10^{2}$ $\left(1.12\times 10^{2}\right)$	**0.94** (**0.06**)	0.26 (0.09)	0.42 (0.08)	0.03 (0.03)	**9.20** (**1.11**)	5.13 (1.78)	7.73 (1.81)	4.47 (1.54)	**0.96** (**0.01**)	0.78 (0.03)	0.88 (0.06)	0.66 (0.03)
	Mean		$2.62\times 10^{5}$ $\left(7.26\times 10^{4}\right)$	$3.60\times 10^{3}$ $\left(1.00\times 10^{3}\right)$	$1.29\times 10^{4}$ $\left(2.27\times 10^{3}\right)$	$1.28\times 10^{3}$ $\left(4.21\times 10^{2}\right)$	**0.83** (**0.1**)	0.36 (0.14)	0.49 (0.13)	0.07 (0.05)	**8.27** (**1.16**)	5.90 (1.30)	7.70 (1.62)	5.75 (1.61)	**0.92** (**0.02**)	0.86 (0.03)	0.89 (0.04)	0.75 (0.04)

**Table 3:** The estimated difference in the average performance between the row and column algorithms for the metrics execution time, coverage, cardinality, and hypervolume. Only results statistically significant at 95% were calculated (ns, no statistically significant result). For the execution time metric, negative values indicate superiority for the algorithms in the rows. For the other metrics, negative values indicate superiority for the algorithms in the columns.

	Execution Time			Coverage			Cardinality			Hypervolume		
	EMR	EMAS	EMNF	EMR	EMAS	EMNF	EMR	EMAS	EMNF	EMR	EMAS	EMNF
EMAS	−87,496	-	-	−0.41	-	-	−1.6	-	-	ns	-	-
EMNF	−80,959	ns	-	ns	ns	-	ns	ns	-	ns	ns	-
WEM	−89,594	−187.8	−1112.5	–0.73	ns	−0.46	−2.2	ns	−1.7	−0.17	−0.10	−0.12
